# Bifurcations and optimal control in Nipah virus epidemiology

**DOI:** 10.1371/journal.pone.0342764

**Published:** 2026-03-11

**Authors:** Zasmin Haque, Md. Mashih Ibn Yasin Adan, Md. Sabab Zulfiker, Faizunnesa Khondaker, Md. Kamrujjaman

**Affiliations:** 1 Department of Mathematics, University of Dhaka, Dhaka, Bangladesh; 2 Department of Mathematics, American International University-Bangladesh, Dhaka, Bangladesh; 3 Department of Mathematics, Kishoreganj University, Kishoreganj, Bangladesh; 4 Department of Computer Science and Engineering, Kishoreganj University, Kishoreganj, Bangladesh; 5 Department of Mathematics, Jagannath University, Dhaka, Bangladesh; Princess Sumaya University for Technology, JORDAN

## Abstract

Nipah virus (NiV) is a zoonotic pathogen with a high case fatality rate, posing a significant and ongoing threat to public health in Asia. This study develops a comprehensive mathematical framework to analyze its transmission dynamics and evaluate effective control strategies. We introduce a novel six-compartment model (SEAIHR) that stratifies the population into Susceptible, Exposed, Asymptomatic, Symptomatic Infected, Hospitalized, and Recovered individuals, incorporating key features such as waning immunity. Analytical results determine the basic reproduction number and establish the global stability of both the disease-free and disease equilibria, confirming a forward bifurcation at the epidemic threshold. A sensitivity analysis identifies the recruitment rate and the disease transmission rate as the most influential parameters on outbreak potential. Furthermore, we formulate an optimal control problem to evaluate the impact of three time-dependent intervention measures: public health campaigns to reduce contact, isolation of symptomatic individuals, and improved treatment for hospitalized patients. The optimal strategies derived from Pontryagin’s Maximum Principle demonstrate a significant reduction in the overall infection burden and intervention costs. Numerical simulations validate the model and show that these combined controls can effectively minimize the final epidemic size while increasing the population’s immunity. This work provides a quantitative framework to guide the design of efficient public health policies for managing and mitigating Nipah virus outbreaks.

## Highlights

- SEAIHR model for Nipah virus with asymptomatic cases.
- Forward bifurcation at ${ℛ}_{0}=1$ shows manageable outbreak.
- Sensitivity analysis finds recruitment and transmission key.
- Optimal control framework with three interventions.
- New metrics: Final Epidemic Size and Fraction Immunized.
- Simulations show optimized controls reduce outbreak size.

## 1 Introduction

Zoonotic pathogens represent a significant and ongoing threat to global public health, with the Nipah virus (NiV) standing out due to its exceptionally high case fatality rate, which historically ranges between 40% and 75%. A member of the *Paramyxoviridae* family, alongside the closely related Hendra virus, Nipah virus was first identified during outbreaks in Malaysia and Singapore (1998–1999) [1,2]. Initial transmission to humans was primarily linked to direct contact with infected pigs. However, subsequent outbreaks across Bangladesh and India revealed a distinct transmission pathway: direct zoonotic transmission from Pteropid fruit bats (flying foxes) the virus’s natural reservoir to humans through the consumption of food items, such as raw date palm sap, contaminated with bat secretions [3–5]. Bats serve as the natural reservoirs of the virus, facilitating direct spillover events to humans that can initiate outbreaks. By modeling the interactions between bat populations and humans, we can gain insights into the mechanisms of transmission, including environmental factors that may drive spillover events. Additionally, integrating an intermediate-host compartment allows for the inclusion of other species that may play a critical role in the transmission cycle, thereby enhancing our understanding of the virus’s ecology and its pathways within the host population. The clinical presentation of Nipah virus infection is severe, beginning with non-specific influenza-like symptoms (fever, headache, respiratory distress) that can rapidly progress to fatal encephalitis, characterized by disorientation, drowsiness, seizures, and coma [1]. Alarmingly, human-to-human transmission via respiratory droplets has also been documented, heightening the risk of nosocomial outbreaks [6]. The absence of approved vaccines or specific antiviral treatments underscores the critical importance of preventive measures, outbreak containment, and supportive care as the cornerstone of current management strategies [7]. Mathematical modeling has emerged as a powerful tool in epidemiology, providing a quantitative framework to understand the complex dynamics of infectious disease transmission, evaluate the potential impact of control interventions, and guide public health policy. The Nipah virus poses significant public health challenges, as highlighted in previous studies [8,9]. Several mathematical models have been developed to understand its transmission dynamics [10,11].

While several mathematical models have been developed to study the transmission dynamics of Nipah virus (NiV) and related paramyxoviruses such as Hendra virus, measles, and SARS-CoV-2, the present study introduces a novel six-compartment SEAIHR framework that extends existing epidemiological models in several key respects. Earlier NiV models often focused on simplified SEIR or SEIRS structures, which did not explicitly account for asymptomatic transmission, hospitalization dynamics, or waning immunity features that are critical for capturing the unique zoonotic and nosocomial transmission pathways of NiV. Previous studies [12] on Hendra virus and measles typically emphasized direct transmission or vector-mediated spread without integrating hospital-based transmission or differentiated asymptomatic and symptomatic infectious states. Similarly, while SARS-CoV-2 models have extensively incorporated asymptomatic compartments and control measures, they often lack specific structures for bat-to-human spillover and hospital-linked outbreaks, which are central to NiV epidemiology [13].

In contrast to prior NiV models that treated exposed individuals as a homogeneous group, the SEAIHR model stratifies the exposed population into asymptomatic and symptomatic classes, allowing for distinct transmission rates and progression pathways. This stratification enables a more nuanced analysis of hidden transmission from asymptomatic carriers a factor less emphasized in earlier paramyxovirus models. Furthermore, the inclusion of a hospitalized compartment with disease-induced death rates and treatment-driven recovery addresses the high fatality and nosocomial transmission risks documented in NiV outbreaks, a feature not comprehensively modeled in earlier works on Hendra or measles. The model also incorporates waning immunity, a refinement over many existing NiV models that assume lifelong immunity post-recovery. This addition aligns more closely with recent immunological findings on NiV and enhances the model’s utility for long-term outbreak planning. Additionally, the forward bifurcation analysis at ${ℛ}_{0}=1$ and the sensitivity analysis identifying recruitment rate and transmission rate as dominant parameters provide new quantitative insights that were not systematically explored in earlier NiV studies. Finally, the optimal control framework introduced here evaluating time-dependent interventions such as contact reduction, isolation, and enhanced treatment extends the work on control strategies for SARS-CoV-2 and other respiratory viruses to the specific context of NiV, offering a tailored approach for resource-limited settings where NiV remains endemic. By integrating these features, the SEAIHR model provides a more comprehensive and biologically realistic tool for understanding NiV transmission, assessing intervention impacts, and guiding public health policy, thereby filling gaps left by prior paramyxovirus and zoonotic disease models.

This study presents a compartmental model to analyze the transmission dynamics of the Nipah virus, incorporating its distinct zoonotic and anthropogenic pathways. In recent years, Nipah virus (NiV) has emerged as a significant zoonotic threat, with unique transmission pathways that necessitate tailored epidemiological models. This study introduces a six-compartment SEAIHR model that not only extends classical SEIR frameworks but also explicitly incorporates the distinctive epidemiological dynamics of NiV, including bat-to-human spillover, nosocomial (hospital) transmission, and human-to-human amplification. By linking model parameters to these specific pathways, we aim to provide a more comprehensive understanding of Nipah virus transmission and inform effective public health strategies. We undertake a rigorous mathematical evaluation of the proposed model. Through this integrated analytical and numerical approach, this work aims to deepen the understanding of Nipah virus epidemiology and contribute to the development of more effective strategies for its surveillance, prevention, and control. The study is organized into several key sections. Sect 2 provides the background of Nipah virus in Asia, setting the epidemiological context for the work. In sect 3, we formulate the mathematical model, which is further analyzed in Sect 4 through local and global stability analysis of equilibria. Sect 5 explores the presence of forward bifurcation, while Sect 6 presents parameter estimation and sensitivity analysis to identify the most influential factors driving disease dynamics. Sect 7 offers numerical simulations that illustrate the model’s behavior, and Sect 8 introduces the optimal control framework, where different strategies are examined and the concepts of the Fraction of Immunized Agents (FIA) and Final Epidemic Size (FES) are incorporated. Finally, Sect 9 summarizes the findings and provides a discussion of their implications. In the *Supplementary file*, we also establish the well-posedness of the model by proving existence, uniqueness, boundedness, and positivity of solutions, ensuring that the model’s predictions remain mathematically consistent and biologically meaningful.

## 2 Background of Nipah virus in Asia

**Fig 1:**
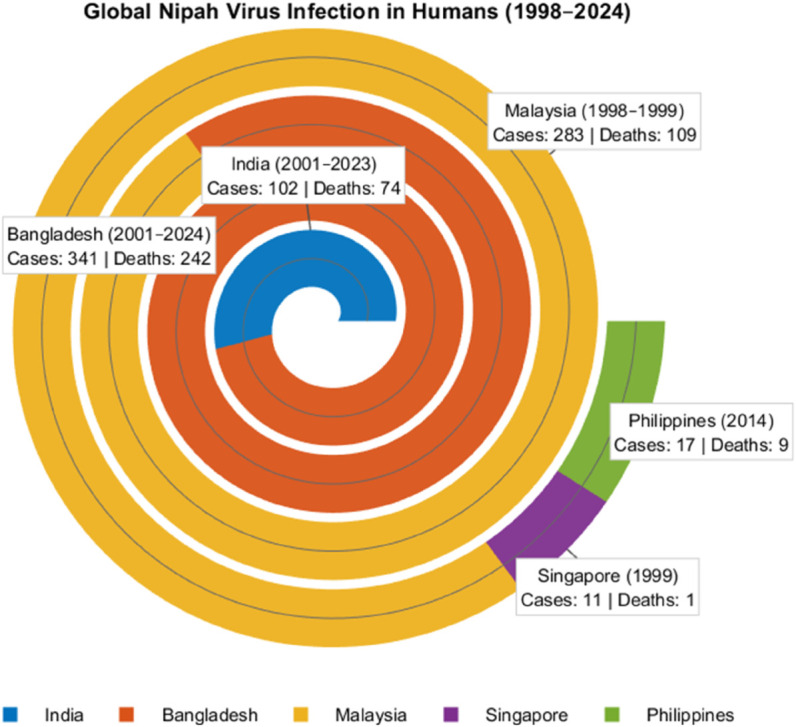
Comparative overview of Nipah virus impact across countries in Asia (1998-2024).

The impact of the Nipah virus has been devastating during past outbreaks, including the 1998-2024 outbreaks across Asia. Fig 1 illustrates the global spread of the Nipah virus [7].

The initial cases of Nipah virus in humans were documented in Bangladesh and India in 2001, with intermittent outbreaks continuing in both nations [6]. Currently, it is believed that the elementary sources of Nipah virus infection in Bangladesh stem from the ingestion of fresh date palm sap and fruits that have been partially consumed and contaminated by the saliva or urine of infected fruit bats. As of 2001, Bangladesh has documented a cumulative total of 341 cases, resulting in 242 deaths, which corresponds to a case mortality rate of 71% across 34 of its 64 districts [6]. During the same period, India has documented 102 cases resulting in 74 deaths, indicating a case mortality rate of 73%, mainly in the states of West Bengal and Kerala. The virus also reached Singapore, where 11 cases and one death were reported following exposure to pigs imported from Malaysia [14]. Between 1998 and 2015, the World Health Organization (WHO) documented a total of more than 600 reported cases, highlighting the significant impact of the disease during this period. Officials indicate that this outbreak represents the sixth Nipah virus spillover event since 2018 and the second occurrence this year. Subsequent outbreaks in Kerala, India, in 2019 and 2021 were managed more effectively, with each incident resulting in only one reported death. In August and September 2023, however, Kerala faced another outbreak, which saw the first patient pass away from the first death attributed to the virus occurred on August 30, subsequently followed by a second fatality that was reported on September 11 [15,16]. Earlier in 2023, Bangladesh encountered a concerning situation, reporting 11 cases and eight fatalities. The country experiences outbreaks almost annually and, in May 2024, a comprehensive review indicates that the highest number of human Nipah virus (NiV) cases globally has been recorded in this region, accounting for 45% of all cases, and 56% of total deaths worldwide which is showed in Fig 1 [6]. The World Health Organization estimates the fatality rate of the virus to be between 40% and 75% [17].

**Fig 2:**
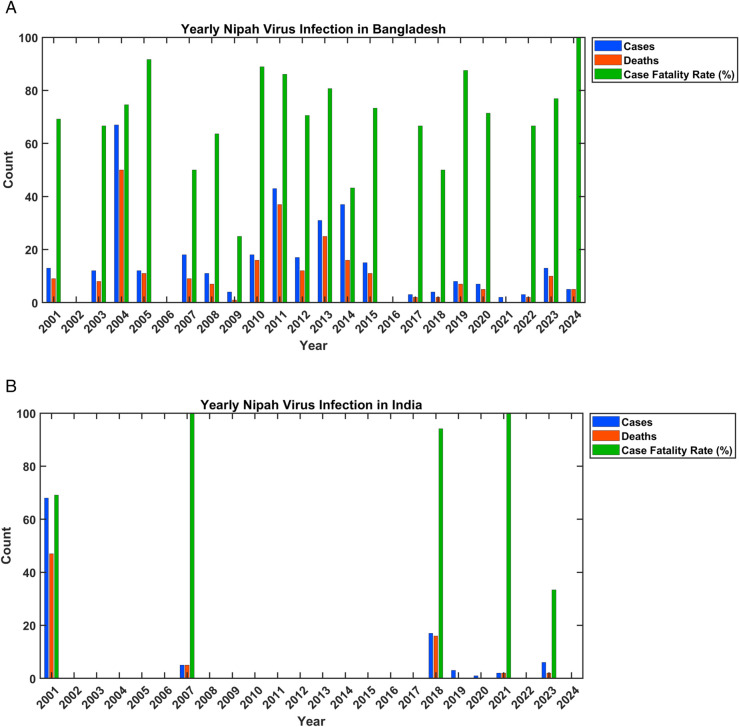
Nipah virus cases, deaths, and fatality rates in Bangladesh and India (2001-2024).

Fig 2 displays the study examines the epidemiological trends in human Nipah virus cases, associated fatalities, and the corresponding case fatality rates (CFR) in Bangladesh (Fig 2(a)) and India (Fig 2(b)), tracing their developments from the year 2001 onward to 2024, based on data collected up to May 31, 2024 [6]. There are no vaccines or treatments that have received approval for use in humans for the Nipah virus, highlighting a significant gap in medical intervention against this disease [7]. Nonetheless, on January 11, 2024, the inaugural human trial of the ChAdOx1 NipahB vaccine was initiated, a groundbreaking development spearheaded by researchers at the University’s Pandemic Sciences Institute [16]. This project is set to continue over the next 18 months, with additional trials anticipated to take place in countries affected by Nipah [18]. Data gathered from the World Health Organization (WHO) plays a crucial role in aiding experts to comprehend the behavior of the Nipah virus and its potential evolution [18]. This information is valuable not only for retrospective analysis but also for predicting future trends and enhancing preparedness and response measures. While Nipah virus has not reached global pandemic levels, its potential for rapid transmission and high mortality rate makes it a serious public health concern. As ongoing research and data analysis reveal more about Nipah virus, we can work toward more effective prevention and treatment strategies. Additionally, this knowledge contributes to our understanding of emerging infectious diseases and their impact on human populations. The Nipah virus remains a significant threat, with outbreaks primarily reported in parts of South and Southeast Asia. Previous literature indicates the effectiveness of various control strategies in managing Nipah virus outbreaks [8]. Additionally, mathematical modeling has emerged as a valuable tool for predicting epidemic dynamics [10,11]. Public health organizations and researchers are working to develop strategies to control and ultimately eliminate the threat of Nipah virus (NiV) and other emerging infectious diseases. This study focuses on a six-compartment mathematical model created to visualize NiV transmission dynamics and evaluate approaches to curb its spread. Biomedical models like this play a critical role in assessing the effectiveness of various intervention strategies, including vaccination campaigns, educate communities, isolate cases, and treatment options. They emphasize the importance of early detection, use protective equipment, practice hygiene, timely isolation of infected individuals, and rigorous contact tracing to efficiently contain outbreaks.

## 3 Mathematical model

A formalized six-compartmental mathematical framework, referred to as SEAIHR, aimed at rigorously analyzing the behavior of the Nipah virus (NiV) pandemic. Whole individuals N(t) is partition into six compartments: the first compartment, S(t), represents the susceptible individuals at time *t*. The model includes an incubation period during which an infected individual remains asymptomatic before becoming infectious, leading to the definition of the exposed class *E*(*t*). The compartments A(t) denote individuals who have the disease but do not exhibit symptoms (asymptomatic). Symptomatic individuals who can transmit the disease but are not hospitalized are represented by I(t), while those who are hospitalized are denoted by H(t). Lastly, R(t) represents individuals who have successfully recovered from the virus. Therefore, the entire individuals at time *t* is


N≡S+E+A+I+H+R.

The force of infection is defined as λ(t)=ρ(A+I), where *ρ* is an effective contact rate that scales with the number of infectious population (asymptomatic and symptomatic). The SEAIHR model was adapted based on techniques outlined in earlier studies [9,11]. The framework is characterized by the subsequent system of differential equations

$$\left\{ \begin{array}{c} \dot{S}=\Lambda +\sigma R-\rho S(A+I)-\mu S,\\ \dot{E}=\rho S(A+I)-(\beta_{1}+\beta_{2}+\mu )E,\\ \dot{A}=\beta_{1}E-(\gamma_{1}+\gamma_{2}+\mu )A,\\ \dot{I}=\beta_{2}E+\gamma_{2}A-(\alpha +\delta_{1}+\mu )I,\\ \dot{H}=\alpha I-(\tau +\delta_{2}+\mu )H,\\ \dot{R}=\gamma_{1}A+\tau H-(\sigma +\mu )R, \end{array}\right.$$ (1)

where the initial conditions S(0)>0, E(0)≥0, A(0)≥0, I(0)≥0, H(0)≥0, R(0)≥0.

**Fig 3:**
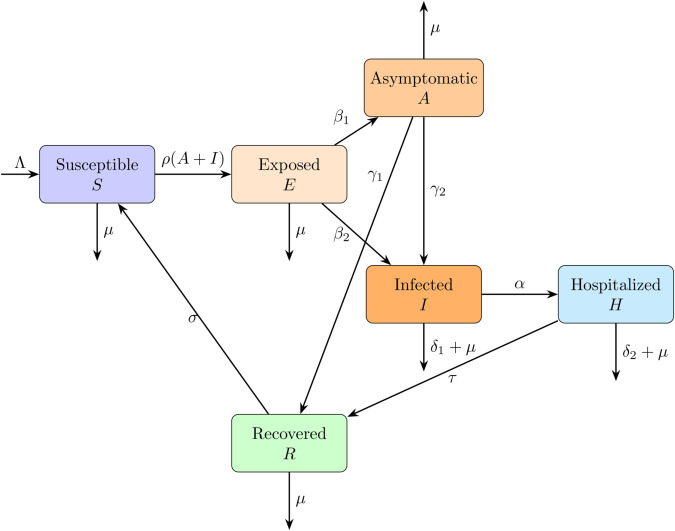
Flow diagram of the SEAIHR model for Nipah virus transmission.

The biological interpretation of all the parameters in the model is that they are assumed to be non-negative numerical values. In contrast to the models presented in [20–23], our model takes into account the movement of asymptomatic exposed population is classified into either the susceptible or infected categories, depending on the severity of the disease manifestation. Additionally, some asymptomatic individuals transition to the recovered class. The model structure is depicted in Fig 3, and the corresponding system parameters are listed in Table 1.

**Table 1:** Description of the parameters of the model (1).

Description	Parameters	Values in days	Reference
Recruitment rate	Λ	0.08	Assumed
Effective contact rate (S→E)	*ρ*	0.45	Estimated
Natural death rate	*μ*	0.0000137	[19]
Transmission rate (E→A)	$\beta_{1}$	0.063	Estimated
Transmission rate (E→I)	$\beta_{2}$	0.055	Estimated
Rate of recovery in *A*	$\gamma_{1}$	0.03	Estimated
Progression to symptomatic	$\gamma_{2}$	0.04	Estimated
Immunity waning rate	*σ*	0.024	Estimated
Hospitalization rate	*α*	0.05	Estimated
Disease-induced death rate in I	$\delta_{1}$	0.031	Estimated
Rate of recovery in *H*	*τ*	0.09	Estimated
Disease-induced death rate in *H*	$\delta_{2}$	0.04	Estimated

The mathematical model is pivotal for the effective management and mitigation of the transmission dynamics associated with the NiV and other infectious diseases. Our model, divided into six compartments, provides a structured framework to analyze disease progression by classifying individuals based on different stages of infection. These compartments may include categories such as susceptible, exposed, infected, recovered, deceased, and possibly vaccinated. By modeling these compartments, researchers can simulate how the virus moves through a population over time, allowing for a more detailed understanding of its transmission dynamics. Such models are instrumental in predicting potential outbreaks and understanding critical factors influencing the virus’s spread. Researchers use this model to evaluate how different stages of infection affect the overall transmission rate, considering variables like the incubation period and infectious period. Additionally, the model allows researchers to simulate various public health interventions. For example, they can examine how timely quarantine measures might reduce infection rates by isolating exposed or infected individuals. Similarly, they can simulate the impact of vaccination campaigns, assessing how immunizing a portion of the population might curb transmission. Through these simulations, the model provides valuable insights into how different strategies might control the Nipah virus’s spread. Each strategy’s simulated outcomes can inform policymakers and health officials about which interventions are most effective. For instance, if a combination of vaccination and quarantine is shown to be highly effective in reducing transmission, this might become the preferred strategy. Ultimately, the model serves as a decision-support tool, aiding public health experts in allocating resources and prioritizing actions that offer the greatest benefit. Such predictive modeling is particularly valuable in regions at high risk for Nipah outbreaks, enabling proactive measures that minimize both health and economic impacts.

## 4 Equilibria and stability: Local and global analysis

This section identifies the disease-free equilibrium (DFE) and the disease equilibrium (DE) of the model (3.1), states the conditions under which each exists, and explains their qualitative behavior. We also clarify how movement between these regimes relates to the basic reproduction number and this implies for persistence, elimination, and control priorities.

### 4.1 Equilibria

#### Disease free equilibrium.

To calculate the disease-free equilibrium of model (3.1), we set the right-hand side of the equations to zero, substituting *E* = 0, *A* = 0, *I* = 0, and *H* = 0; this process yields the following results:


$${𝒟}_{0}=\left(S_{0},0,0,0,0,0\right)=\left(\frac{\Lambda }{\mu },0,0,0,0,0\right).$$

**Theorem 1.** *The model (3.1) yields the basic reproduction number, which is expressed as*


$${ℛ}_{0}=\frac{\rho \Lambda }{\mu }\left(\frac{\beta_{1}(\alpha +\delta_{1}+\mu +\gamma_{2})+\beta_{2}(\gamma_{1}+\gamma_{2}+\mu )}{\left(\beta_{1}+\beta_{2}+\mu )(\gamma_{1}+\gamma_{2}+\mu )(\alpha +\delta_{1}+\mu \right)}\right).$$

*Proof:* In model (3.1), the compartments E,A,I,H represent the infected classes. We can express the right-hand side of the system (3.1) corresponding to these infected compartments as ℱ−𝒱, where


$$ℱ=(\begin{array}{c} \rho S(A+I)\\ 0\\ 0\\ 0 \end{array});\;𝒱=(\begin{array}{c} (\beta_{1}+\beta_{2}+\mu )E\\ -\beta_{1}E+(\gamma_{1}+\gamma_{2}+\mu )A\\ -\beta_{2}E-\gamma_{2}A+(\alpha +\delta_{1}+\mu )I\\ -\alpha I+(\tau +\delta_{2}+\mu )H \end{array}).$$

At this stage, we proceed to evaluate the derivatives of ℱ and 𝒱 at the disease-free equilibrium ${𝒟}_{0}$. This evaluation yields two matrices, denoted as *F* (transmission terms) and V (transition terms), where


$$F=(\begin{array}{cccc} 0 & \rho S_{0} & \rho S_{0} & 0\\ 0 & 0 & 0 & 0\\ 0 & 0 & 0 & 0\\ 0 & 0 & 0 & 0 \end{array});\;\;V=(\begin{array}{cccc} \left(\beta_{1}+\beta_{2}+\mu \right) & 0 & 0 & 0\\ -\beta_{1} & \left(\gamma_{1}+\gamma_{2}+\mu \right) & 0 & 0\\ -\beta_{2} & -\gamma_{2} & \left(\alpha +\delta_{1}+\mu \right) & 0\\ 0 & 0 & -\alpha & \left(\tau +\delta_{2}+\mu \right) \end{array}).$$

Thus, the inverse matrix of *V* is


$$V^{-1}=(\begin{array}{cccc} \frac{1}{k_{1}} & 0 & 0 & 0\\ \frac{\beta_{1}}{k_{1}k_{2}} & \frac{1}{k_{2}} & 0 & 0\\ \frac{\beta_{2}k_{2}+\beta_{1}\gamma_{2}}{k_{1}k_{2}k_{3}} & \frac{\gamma_{2}}{k_{2}k_{3}} & \frac{1}{k_{3}} & 0\\ \frac{\alpha (\beta_{2}k_{2}+\beta_{1}\gamma_{2})}{k_{1}k_{2}k_{3}k_{4}} & \frac{\alpha \gamma_{2}}{k_{2}k_{3}k_{4}} & \frac{\alpha }{k_{3}k_{4}} & \frac{1}{k_{4}} \end{array}),$$

where, $k_{1}=\beta_{1}+\beta_{2}+\mu ,\;k_{2}=\gamma_{1}+\gamma_{2}+\mu ,\;k_{3}=\alpha +\delta_{1}+\mu ,\;k_{4}=\tau +\delta_{2}+\mu$.

Consequently, the next generation matrix [24] can be expressed as


$$FV^{-1}=(\begin{array}{cccc} \frac{\rho S_{0}\beta_{1}}{k_{1}k_{2}}+\frac{\rho S_{0}(\beta_{2}k_{2}+\beta_{1}\gamma_{2})}{k_{1}k_{2}k_{3}} & \frac{\rho S_{0}}{k_{2}}+\frac{\rho S_{0}\gamma_{2}}{k_{2}k_{3}} & \frac{\rho S_{0}}{k_{3}} & 0\\ 0 & 0 & 0 & 0\\ 0 & 0 & 0 & 0\\ 0 & 0 & 0 & 0 \end{array}).$$

By determining the dominant eigenvalue of the next generation matrix $FV^{-1}$, we obtain the basic reproduction number, which is given by


$${ℛ}_{0}=\frac{\rho S_{0}\beta_{1}}{k_{1}k_{2}}+\frac{\rho S_{0}(\beta_{2}k_{2}+\beta_{1}\gamma_{2})}{k_{1}k_{2}k_{3}}=\rho S_{0}\left(\frac{\beta_{1}}{k_{1}k_{2}}+\frac{\beta_{2}}{k_{1}k_{3}}+\frac{\beta_{1}\gamma_{2}}{k_{1}k_{2}k_{3}}\right).$$

Hence, by substituting the values of $S_{0},k_{1},k_{2},$ and *k*_3_ into the expression, we simplify it as follows:

$${ℛ}_{0}=\frac{\rho \Lambda }{\mu }\left(\frac{\beta_{1}(\alpha +\delta_{1}+\mu )+\beta_{2}(\gamma_{1}+\gamma_{2}+\mu )+\beta_{1}\gamma_{2}}{\left(\beta_{1}+\beta_{2}+\mu )(\gamma_{1}+\gamma_{2}+\mu )(\alpha +\delta_{1}+\mu \right)}\right).$$

$$∴{ℛ}_{0}=\frac{\rho \Lambda }{\mu }\left(\frac{\beta_{1}(\alpha +\delta_{1}+\mu +\gamma_{2})+\beta_{2}(\gamma_{1}+\gamma_{2}+\mu )}{\left(\beta_{1}+\beta_{2}+\mu )(\gamma_{1}+\gamma_{2}+\mu )(\alpha +\delta_{1}+\mu \right)}\right).$$ (2)

□

Here, the basic reproduction number ${ℛ}_{0}$ for the SEAIHR model quantifies the average number of secondary Nipah virus infections generated by a single infectious individual in a fully susceptible population. When ${ℛ}_{0}<1$, the disease dies out; when ${ℛ}_{0}>1$, an outbreak can occur and become endemic. The derived expression ${ℛ}_{0}=\frac{\rho \Lambda }{\mu }\left(\frac{\beta_{1}(\alpha +\delta_{1}+\mu +\gamma_{2})+\beta_{2}(\gamma_{1}+\gamma_{2}+\mu )}{\left(\beta_{1}+\beta_{2}+\mu )(\gamma_{1}+\gamma_{2}+\mu )(\alpha +\delta_{1}+\mu \right)}\right)$ incorporates contributions from both asymptomatic ($\beta_{1}$) and symptomatic ($\beta_{2}$) transmission pathways, moderated by progression, hospitalization, recovery, and death rates.

#### Disease equilibrium.

The endemic equilibrium is obtained by setting all time derivatives to zero $\left(\dot{S}=\dot{E}=\dot{A}=\dot{I}=\dot{H}=\dot{R}=0\right)$ and solving the resulting system of equations. Below are the expressions for the endemic equilibrium points:


$$\left\{ \begin{array}{c} S^{*}=\frac{(\beta_{1}+\beta_{2}+\mu )m_{1}m_{2}}{\rho (\beta_{1}m_{2}+\beta_{2}m_{1}+\gamma_{2}\beta_{1})},\\ E^{*}=\frac{\Lambda \left(\beta_{1}m_{2}+\beta_{2}m_{1}+\gamma_{2}\beta_{1}\right)-\mu (\beta_{1}+\beta_{2}+\mu )m_{1}m_{2}}{\rho (\beta_{1}+\beta_{2}+\mu )m_{1}m_{2}-\beta_{1}m_{2}(\beta_{1}m_{2}+\beta_{2}m_{1}+\gamma_{2}\beta_{1})-\sigma \left(\gamma_{1}\beta_{1}m_{2}m_{3}+\tau \alpha (\beta_{2}m_{1}+\gamma_{2}\beta_{1})\right)},\\ A^{*}=\frac{\beta_{1}}{m_{1}}E^{*},\\ I^{*}=\frac{\beta_{2}m_{1}+\gamma_{2}\beta_{1}}{m_{1}m_{2}}E^{*},\\ H^{*}=\frac{\alpha (\beta_{2}m_{1}+\gamma_{2}\beta_{1})}{m_{1}m_{2}m_{3}}E^{*},\\ R^{*}=\frac{\gamma_{1}\beta_{1}m_{2}m_{3}+\tau \alpha (\beta_{2}m_{1}+\gamma_{2}\beta_{1})}{m_{1}m_{2}m_{3}m_{4}}E^{*}, \end{array}\right.$$

where, $m_{1}=\gamma_{1}+\gamma_{2}+\mu ,\;m_{2}=\alpha +\delta_{1}+\mu ,\;m_{3}=\tau +\delta_{2}+\mu ,\;m_{4}=\sigma +\mu .$

**Remark 1.** *If* $\Lambda \left(\beta_{1}m_{2}+\beta_{2}m_{1}+\gamma_{2}\beta_{1}\right)>\mu (\beta_{1}+\beta_{2}+\mu )m_{1}m_{2}$, then E^*^ > 0.

### 4.2 Local stability analysis of DFE and DE

Initially, we will perform a local stability analysis of the Disease-Free Equilibrium (DFE), subsequently addressing the stability of the Disease Equilibrium (DE). It is noteworthy that the proof of Theorem 2 is analogous to that in [[20], Theorem 5].

**Theorem 2.** *The condition for local asymptotic stability of the Disease-Free Equilibrium ${𝒟}_{0}$ of model (3.1) is ${ℛ}_{0}<1$; conversely, it is deemed unstable when ${ℛ}_{0}>1$.*

*Proof:* The local stability of the Disease-Free Equilibrium (DFE) ${𝒟}_{0}$ is assessed by examining the eigenvalues of the Jacobian matrix at ${𝒟}_{0}$, which is expressed as follows:


$$J_{\text{DFE}}=(\begin{array}{cccccc} -\mu & 0 & -\rho \frac{\Lambda }{\mu } & -\rho \frac{\Lambda }{\mu } & 0 & \sigma \\ 0 & -(\beta_{1}+\beta_{2}+\mu ) & \rho \frac{\Lambda }{\mu } & \rho \frac{\Lambda }{\mu } & 0 & 0\\ 0 & \beta_{1} & -(\gamma_{1}+\gamma_{2}+\mu ) & 0 & 0 & 0\\ 0 & \beta_{2} & \gamma_{2} & -(\alpha +\delta_{1}+\mu ) & 0 & 0\\ 0 & 0 & 0 & \alpha & -(\tau +\delta_{2}+\mu ) & 0\\ 0 & 0 & \gamma_{1} & 0 & \tau & -(\sigma +\mu ) \end{array}).$$

The eigenvalues *λ* are found by solving $\det(J_{\text{DFE}}-\lambda I)=0$. Due to the block-triangular structure, the eigenvalues decompose into two sets:

1. Non-Infected Compartments (S,R):
  $$|\begin{array}{cc} -\mu -\lambda & \sigma \\ 0 & -(\sigma +\mu )-\lambda \end{array}|=(-\mu -\lambda )(-(\sigma +\mu )-\lambda )=0.$$
  Eigenvalues:
  $$\lambda_{1}=-\mu ,\;\lambda_{2}=-(\sigma +\mu ).$$
  Both are always negative, implying stability in *S* and *R*.
2. Infected Compartments (E,A,I,H): The submatrix for E,A,I,H is:
  $$J_{\text{infected}}=(\begin{array}{cccc} -(\beta_{1}+\beta_{2}+\mu ) & \rho \frac{\Lambda }{\mu } & \rho \frac{\Lambda }{\mu } & 0\\ \beta_{1} & -(\gamma_{1}+\gamma_{2}+\mu ) & 0 & 0\\ \beta_{2} & \gamma_{2} & -(\alpha +\delta_{1}+\mu ) & 0\\ 0 & 0 & \alpha & -(\tau +\delta_{2}+\mu ) \end{array}).$$
  The characteristic equation is:
  $$\det(J_{\text{infected}}-\lambda I)=0.$$
  This is a quartic equation in *λ*:
  $$P(\lambda )=\lambda^{4}+a_{1}\lambda^{3}+a_{2}\lambda^{2}+a_{3}\lambda +a_{4}=0.$$

To determine the eigenvalues from the characteristic equation $\det(J_{\text{infected}}-\lambda I)=0$, we analyze the infected subsystem Jacobian matrix:


$$J_{\text{infected}}=(\begin{array}{cccc} -(\beta_{1}+\beta_{2}+\mu ) & \rho \frac{\Lambda }{\mu } & \rho \frac{\Lambda }{\mu } & 0\\ \beta_{1} & -(\gamma_{1}+\gamma_{2}+\mu ) & 0 & 0\\ \beta_{2} & \gamma_{2} & -(\alpha +\delta_{1}+\mu ) & 0\\ 0 & 0 & \alpha & -(\tau +\delta_{2}+\mu ) \end{array}).$$

The characteristic equation is derived from $\det(J_{\text{infected}}\;-\;\lambda I)=0$, where *I* is the identity matrix. The determinant of $J_{\text{infected}}-\lambda I$ expands to:


$$P(\lambda )=|\begin{array}{cccc} -(\beta_{1}+\beta_{2}+\mu )-\lambda & \rho \frac{\Lambda }{\mu } & \rho \frac{\Lambda }{\mu } & 0\\ \beta_{1} & -(\gamma_{1}+\gamma_{2}+\mu )-\lambda & 0 & 0\\ \beta_{2} & \gamma_{2} & -(\alpha +\delta_{1}+\mu )-\lambda & 0\\ 0 & 0 & \alpha & -(\tau +\delta_{2}+\mu )-\lambda \end{array}|.$$

Using block matrix properties (due to lower-triangular structure), this simplifies to:


$$P(\lambda )=\left(-(\tau +\delta_{2}+\mu )-\lambda \right)\cdot \det(M),$$

where *M* is the 3×3 submatrix:


$$M=(\begin{array}{ccc} -(\beta_{1}+\beta_{2}+\mu )-\lambda & \rho \frac{\Lambda }{\mu } & \rho \frac{\Lambda }{\mu }\\ \beta_{1} & -(\gamma_{1}+\gamma_{2}+\mu )-\lambda & 0\\ \beta_{2} & \gamma_{2} & -(\alpha +\delta_{1}+\mu )-\lambda \end{array}).$$

The eigenvalues split into two parts:

1. From the lower-right block:
  $$\lambda_{1}=-(\tau +\delta_{2}+\mu ).$$
  This is always negative (stable).
2. From the 3×3 submatrix *M*: The remaining eigenvalues $\lambda_{2},\lambda_{3},\lambda_{4}$ are roots of the cubic equation:
  det(M)=0.
  Explicitly:
  $$|\begin{array}{ccc} -(\beta_{1}+\beta_{2}+\mu )-\lambda & \rho \frac{\Lambda }{\mu } & \rho \frac{\Lambda }{\mu }\\ \beta_{1} & -(\gamma_{1}+\gamma_{2}+\mu )-\lambda & 0\\ \beta_{2} & \gamma_{2} & -(\alpha +\delta_{1}+\mu )-\lambda \end{array}|=0.$$
  This simplifies to the cubic equation:
  $$\lambda^{3}+a\lambda^{2}+b\lambda +c=0,$$
  where the coefficients *a*, *b*, and *c* are:
  $$a=(\beta_{1}+\beta_{2}+\mu )+(\gamma_{1}+\gamma_{2}+\mu )+(\alpha +\delta_{1}+\mu ),\;b=(\beta_{1}+\beta_{2}+\mu )(\gamma_{1}+\gamma_{2}+\mu )+(\beta_{1}+\beta_{2}+\mu )(\alpha +\delta_{1}+\mu )\;+(\gamma_{1}+\gamma_{2}+\mu )(\alpha +\delta_{1}+\mu )-\frac{\rho \Lambda }{\mu }(\beta_{1}+\beta_{2}),\;c=(\beta_{1}+\beta_{2}+\mu )(\gamma_{1}+\gamma_{2}+\mu )(\alpha +\delta_{1}+\mu )(1-{ℛ}_{0}).$$

Positivity of *a* and *c* is evident, as *a* > 0 since it represents the sum of positive rates. Additionally, *c* > 0 holds when ${ℛ}_{0}<1$ because $1-{ℛ}_{0}>0$. Regarding the sign of *b*, while the term $-\rho \frac{\Lambda }{\mu }(\beta_{1}+\beta_{2})$ is negative, the remaining terms in *b* are positive.

For the cubic polynomial $P(\lambda )=\lambda^{3}+a\lambda^{2}+b\lambda +c$, the Routh-Hurwitz stability conditions [25–27] require all coefficients to be positive: *a* > 0, *b* > 0, and *c* > 0, along with the inequality *ab* > *c*. We analyze these conditions for both ${ℛ}_{0}<1$ and ${ℛ}_{0}>1$.

The condition *c* > 0 is satisfied since $1-{ℛ}_{0}>0$, and *a* > 0 is always true as it is the sum of positive rates. For *b* > 0, the negative term $-\rho \frac{\Lambda }{\mu }(\beta_{1}+\beta_{2})$ is dominated by the positive terms when ${ℛ}_{0}<1$, as $\rho \frac{\Lambda }{\mu }$ scales with ${ℛ}_{0}$. Specifically, substituting ${ℛ}_{0}$ into *b* yields:


$$b=\text{Positive terms}-\rho \frac{\Lambda }{\mu }(\beta_{1}+\beta_{2})>0\;\text{for small }\rho \frac{\Lambda }{\mu }\text{ (i.e., }{ℛ}_{0}<1).$$

The inequality *ab* > *c* holds since *a* > 0 and *b* > 0, and *c* is proportional to $\left(1-{ℛ}_{0}\right)$, which is valid when ${ℛ}_{0}$ is sufficiently small. In contrast, when ${ℛ}_{0}>1$, *c* < 0 since $1\;-\;{ℛ}_{0}<0$. According to the Routh-Hurwitz criterion, any negative coefficient indicates instability in the system.

To address the concern regarding the negative term in *b*, we can express *b* in terms of ${ℛ}_{0}$. From the definition of (4.1), we have:


$${ℛ}_{0}=\frac{\rho \Lambda \left[\beta_{1}(\alpha +\delta_{1}+\mu +\gamma_{2})+\beta_{2}(\gamma_{1}+\gamma_{2}+\mu )\right]}{\mu (\beta_{1}+\beta_{2}+\mu )(\gamma_{1}+\gamma_{2}+\mu )(\alpha +\delta_{1}+\mu )}.$$

Isolating $\rho \frac{\Lambda }{\mu }$, we find:


$$\rho \frac{\Lambda }{\mu }=\frac{{ℛ}_{0}(\beta_{1}+\beta_{2}+\mu )(\gamma_{1}+\gamma_{2}+\mu )(\alpha +\delta_{1}+\mu )}{\beta_{1}(\alpha +\delta_{1}+\mu +\gamma_{2})+\beta_{2}(\gamma_{1}+\gamma_{2}+\mu )}.$$

Substituting this expression back into *b* gives:


$$b=\text{Positive terms}-\frac{{ℛ}_{0}(\beta_{1}+\beta_{2}+\mu )(\gamma_{1}+\gamma_{2}+\mu )(\alpha +\delta_{1}+\mu )}{\beta_{1}(\alpha +\delta_{1}+\mu +\gamma_{2})+\beta_{2}(\gamma_{1}+\gamma_{2}+\mu )}(\beta_{1}+\beta_{2}).$$

For ${ℛ}_{0}<1$, the negative term is sufficiently small to ensure *b* > 0. Thus, when ${ℛ}_{0}<1$, all Routh-Hurwitz conditions are satisfied, indicating a stable DFE. However, for ${ℛ}_{0}>1$, the condition *c* < 0 violates the Routh-Hurwitz criteria, resulting in an unstable DFE. □

The following theorem establishes that when ${ℛ}_{0}>1$, the disease equilibrium points are locally asymptotically stable. Note that the proof of Theorem 3 is similar to that in [[22], Theorem 2].

**Theorem 3.** *The disease equilibrium points given by (3.1) and $N^{*}=(S^{*},E^{*},A^{*},I^{*},H^{*},R^{*})$ are locally asymptotically stable if ${ℛ}_{0}>1$.*

*Proof:* The Jacobian matrix at the disease equilibrium point $\left(S^{*},E^{*},A^{*},I^{*},H^{*},R^{*}\right)$ for the given model is:


$$J=(\begin{array}{cccccc} -\rho (A^{*}+I^{*})-\mu & 0 & -\rho S^{*} & -\rho S^{*} & 0 & \sigma \\ \rho (A^{*}+I^{*}) & -(\beta_{1}+\beta_{2}+\mu ) & \rho S^{*} & \rho S^{*} & 0 & 0\\ 0 & \beta_{1} & -(\gamma_{1}+\gamma_{2}+\mu ) & 0 & 0 & 0\\ 0 & \beta_{2} & \gamma_{2} & -(\alpha +\delta_{1}+\mu ) & 0 & 0\\ 0 & 0 & 0 & \alpha & -(\tau +\delta_{2}+\mu ) & 0\\ 0 & 0 & \gamma_{1} & 0 & \tau & -(\sigma +\mu ) \end{array}).$$

Substituting the equilibrium values $S^{*},A^{*},I^{*}$ and simplifying the constants $m_{1},m_{2},m_{3},m_{4}$, the Jacobian matrix becomes:


$$J=(\begin{array}{cccccc} -\rho \left(\frac{\beta_{1}}{m_{1}}+\frac{\beta_{2}m_{1}+\gamma_{2}\beta_{1}}{m_{1}m_{2}}\right)E^{*}-\mu & 0 & -\rho S^{*} & -\rho S^{*} & 0 & \sigma \\ \rho \left(\frac{\beta_{1}}{m_{1}}+\frac{\beta_{2}m_{1}+\gamma_{2}\beta_{1}}{m_{1}m_{2}}\right)E^{*} & -(\beta_{1}+\beta_{2}+\mu ) & \rho S^{*} & \rho S^{*} & 0 & 0\\ 0 & \beta_{1} & -m_{1} & 0 & 0 & 0\\ 0 & \beta_{2} & \gamma_{2} & -m_{2} & 0 & 0\\ 0 & 0 & 0 & \alpha & -m_{3} & 0\\ 0 & 0 & \gamma_{1} & 0 & \tau & -m_{4} \end{array}).$$

To determine the eigenvalues of the Jacobian matrix at the disease equilibrium, we solve the characteristic equation det(J−λI)=0, where *J* is the Jacobian matrix and *λ* is an eigenvalue. The Jacobian matrix is:


$$J=(\begin{array}{cccccc} a_{11} & 0 & a_{13} & a_{14} & 0 & \sigma \\ a_{21} & a_{22} & \rho S^{*} & \rho S^{*} & 0 & 0\\ 0 & \beta_{1} & -m_{1} & 0 & 0 & 0\\ 0 & \beta_{2} & \gamma_{2} & -m_{2} & 0 & 0\\ 0 & 0 & 0 & \alpha & -m_{3} & 0\\ 0 & 0 & \gamma_{1} & 0 & \tau & -m_{4} \end{array}),$$

where, $a_{11}=-\rho (A^{*}+I^{*})-\mu ,\;a_{13}=-\rho S^{*},\;a_{14}=-\rho S^{*},\;a_{21}=\rho (A^{*}+I^{*}),\;a_{22}=-(\beta_{1}+\beta_{2}+\mu ).$ The characteristic polynomial is obtained from det(J−λI)=0, where *I* is the identity matrix. The matrix J−λI is:


$$J-\lambda I=(\begin{array}{cccccc} a_{11}-\lambda & 0 & a_{13} & a_{14} & 0 & \sigma \\ a_{21} & a_{22}-\lambda & \rho S^{*} & \rho S^{*} & 0 & 0\\ 0 & \beta_{1} & -m_{1}-\lambda & 0 & 0 & 0\\ 0 & \beta_{2} & \gamma_{2} & -m_{2}-\lambda & 0 & 0\\ 0 & 0 & 0 & \alpha & -m_{3}-\lambda & 0\\ 0 & 0 & \gamma_{1} & 0 & \tau & -m_{4}-\lambda \end{array}).$$

The determinant of a 6×6 matrix is complex; however, due to its block structure, we can simplify the calculation. Since the matrix is in lower block-triangular form, the determinant can be expressed as the product of the determinants of the diagonal blocks. We will adopt a general approach to explore this further. The characteristic equation is:


$$\det(J-\lambda I)=|\begin{array}{cccccc} a_{11}-\lambda & 0 & a_{13} & a_{14} & 0 & \sigma \\ a_{21} & a_{22}-\lambda & \rho S^{*} & \rho S^{*} & 0 & 0\\ 0 & \beta_{1} & -m_{1}-\lambda & 0 & 0 & 0\\ 0 & \beta_{2} & \gamma_{2} & -m_{2}-\lambda & 0 & 0\\ 0 & 0 & 0 & \alpha & -m_{3}-\lambda & 0\\ 0 & 0 & \gamma_{1} & 0 & \tau & -m_{4}-\lambda \end{array}|=0.$$

The matrix can be partitioned into blocks. Let


$$J-\lambda I=(\begin{array}{cc} A & B\\ C & D \end{array}),$$

where,


$$A=(\begin{array}{cc} a_{11}-\lambda & 0\\ a_{21} & a_{22}-\lambda \end{array}),\;B=(\begin{array}{cccc} a_{13} & a_{14} & 0 & \sigma \\ \rho S^{*} & \rho S^{*} & 0 & 0 \end{array}),$$

$$C=(\begin{array}{cc} 0 & \beta_{1}\\ 0 & \beta_{2}\\ 0 & 0\\ 0 & 0 \end{array}),\;D=(\begin{array}{cccc} -m_{1}-\lambda & 0 & 0 & 0\\ \gamma_{2} & -m_{2}-\lambda & 0 & 0\\ 0 & \alpha & -m_{3}-\lambda & 0\\ \gamma_{1} & 0 & \tau & -m_{4}-\lambda \end{array}).$$

The determinant is:


$$\det(J-\lambda I)=\det(A)\cdot \det(D-CA^{-1}B).$$

However, this approach is computationally intensive. Instead, we note that the matrix possesses a lower block structure, allowing us to partition the eigenvalues for a more efficient analysis.

The eigenvalues are derived from the diagonal blocks. The block *D* is lower triangular, so its eigenvalues are its diagonal elements:


$$\lambda_{3}=-m_{1}-\lambda ,\;\lambda_{4}=-m_{2}-\lambda ,\;\lambda_{5}=-m_{3}-\lambda ,\;\lambda_{6}=-m_{4}-\lambda .$$

The remaining eigenvalues come from the 2×2 block *A*:


$$\det(A)=|\begin{array}{cc} a_{11}-\lambda & 0\\ a_{21} & a_{22}-\lambda \end{array}|=(a_{11}-\lambda )(a_{22}-\lambda )=0.$$

Combining all, the six eigenvalues are $\lambda_{1}=a_{11},\;\lambda_{2}=a_{22},\;\lambda_{3}=-m_{1},\;\lambda_{4}=-m_{2},\;\lambda_{5}=-m_{3},\;\lambda_{6}=-m_{4}.$ For stability, all eigenvalues must have negative real parts. The eigenvalues $\lambda_{3},\lambda_{4},\lambda_{5},\lambda_{6}$ are clearly negative since $m_{1},m_{2},m_{3},m_{4}>0$. The stability depends on $\lambda_{1}$ and $\lambda_{2}$. Since $\lambda_{1}=a_{11}=-\rho (A^{*}+I^{*})-\mu$ and $\lambda_{2}=a_{22}=-(\beta_{1}+\beta_{2}+\mu )$, both eigenvalues are negative if $\rho (A^{*}+I^{*})+\mu >0\;\text{and}\;\beta_{1}+\beta_{2}+\mu >0.$ These inequalities always hold because all parameters $\left(\rho ,\mu ,\beta_{1},\beta_{2}\right)$ and equilibrium values $\left(A^{*},I^{*}\right)$ are positive.

Hence, the disease equilibrium N^*^ is locally asymptotically stable whenever it exists (i.e., when ${ℛ}_{0}>1$), as all eigenvalues of the Jacobian matrix at N^*^ have negative real parts. □

### 4.3 Global stability analysis of DFE and DE

We will begin by conducting a global stability analysis of the Disease-Free Equilibrium (DFE) and then proceed to examine the stability of the Disease Equilibrium (DE). Note that the proof of the Theorem 4 is similar to that in [[22], Theorem 3].

**Theorem 4.** *Since ${ℛ}_{0}\leq 1$, the Disease-Free Equilibrium (DFE) of the model (3.1) is globally asymptotically stable in the positively invariant region Ω defined in supplementary file.*

*Proof:* Consider the Lyapunov function defined as:


$$𝒱(S,E,A,I,H,R)=\left(S-S_{0}-S_{0}\ln\frac{S}{S_{0}}\right)+E+A+I+H+R,$$

where, $S_{0}=\frac{\Lambda }{\mu }$.

Differentiating 𝒱 with respect to time along the solutions of the system:


$$\dot{𝒱}=\left(1-\frac{S_{0}}{S}\right)\dot{S}+\dot{E}+\dot{A}+\dot{I}+\dot{H}+\dot{R}.$$

Substituting the expressions for $\dot{S},\dot{E},\dot{A},\dot{I},\dot{H},\dot{R}$ from the given system:


$$\dot{𝒱}=\left(1-\frac{S_{0}}{S}\right)\left(\Lambda +\sigma R-\rho S(A+I)-\mu S\right)+\left(\rho S(A+I)-(\beta_{1}+\beta_{2}+\mu )E\right)\;\;+\left(\beta_{1}E-(\gamma_{1}+\gamma_{2}+\mu )A\right)+\left(\beta_{2}E+\gamma_{2}A-(\alpha +\delta_{1}+\mu )I\right)\;\;+\left(\alpha I-(\tau +\delta_{2}+\mu )H\right)+\left(\gamma_{1}A+\tau H-(\sigma +\mu )R\right).$$

Expanding and simplifying the terms


$$\dot{𝒱}=\Lambda -\mu S-\frac{\Lambda S_{0}}{S}+\mu S_{0}-\rho S(A+I)+\frac{\rho S_{0}(A+I)}{S}+\sigma R\left(1-\frac{S_{0}}{S}\right)+\rho S(A+I)\;\;-(\beta_{1}+\beta_{2}+\mu )E+\beta_{1}E-(\gamma_{1}+\gamma_{2}+\mu )A+\beta_{2}E+\gamma_{2}A-(\alpha +\delta_{1}+\mu )I\;\;+\alpha I-(\tau +\delta_{2}+\mu )H+\gamma_{1}A+\tau H-(\sigma +\mu )R.$$

After canceling out the terms and grouping the remaining terms:


$$\dot{𝒱}=\Lambda -\mu S-\frac{\Lambda S_{0}}{S}+\mu S_{0}+\frac{\rho S_{0}(A+I)}{S}+\sigma R\left(1-\frac{S_{0}}{S}\right)\;\;-\mu E-\mu A-\mu I-\mu H-\mu R-\sigma R-\delta_{1}I-\delta_{2}H.$$

Using $S_{0}=\frac{\Lambda }{\mu }$, we have $\Lambda =\mu S_{0}$. Substituting this:


$$\dot{𝒱}=\mu S_{0}-\mu S-\mu S_{0}\frac{S_{0}}{S}+\mu S_{0}+\frac{\rho S_{0}(A+I)}{S}+\sigma R\left(1-\frac{S_{0}}{S}\right)\;\;-\mu (E+A+I+H+R)-\sigma R-\delta_{1}I-\delta_{2}H.$$

Further simplifying:


$$\dot{𝒱}=\mu S_{0}\left(2-\frac{S}{S_{0}}-\frac{S_{0}}{S}\right)+\frac{\rho S_{0}(A+I)}{S}+\sigma R\left(1-\frac{S_{0}}{S}\right)\;\;-\mu (E+A+I+H+R)-\sigma R-\delta_{1}I-\delta_{2}H.$$

1. The term $\mu S_{0}\left(2-\frac{S}{S_{0}}-\frac{S_{0}}{S}\right)$ can be rewritten as:
  $$-\mu S_{0}\left(\frac{S}{S_{0}}+\frac{S_{0}}{S}-2\right)=-\mu S_{0}\left(\frac{(S-S_{0}{)}^{2}}{SS_{0}}\right)\leq 0,$$
  since $\frac{(S-S_{0}{)}^{2}}{SS_{0}}\geq 0$.
2. The remaining terms involve A,I,H,R. For the DFE to be stable, these terms must be non-positive. When ${ℛ}_{0}\leq 1$, the infected compartments E,A,I,H,R will naturally decay to zero over time, ensuring that the additional terms are non-positive.

Since $\dot{𝒱}\leq 0$ and $\dot{𝒱}=0$ only when *S* = *S*_0_ and *E* = *A* = *I* = *H* = *R* = 0, the largest invariant set in $\left\{(S,E,A,I,H,R)\in \Omega :\dot{𝒱}=0\right\}$ is ${𝒟}_{0}=\left\{\left(\frac{\Lambda }{\mu },0,0,0,0,0\right)\right\}$. By LaSalle’s Invariance Principle, the disease-free equilibrium (DFE) is globally asymptotically stable in the region Ω when ${ℛ}_{0}\leq 1$. □

Now, we establish the global stability of the disease equilibrium (DE). Note that the proof of Theorem 5 is similar to that in [[22], Theorem 4].

**Theorem 5.** *Since ${ℛ}_{0}>1$, the disease equilibrium (DE) given by $N^{*}=(S^{*},E^{*},A^{*},I^{*},H^{*},R^{*})$ of the model (3.1) is globally asymptotically stable in the region Ω defined by Eq (B.1) in Lemma 2 of the Supplementary file.*

*Proof:* We define the Lyapunov function ℒ as:


$$ℒ(S,E,A,I,H,R)=\left(S-S^{*}-S^{*}\ln\frac{S}{S^{*}}\right)+\left(E-E^{*}-E^{*}\ln\frac{E}{E^{*}}\right)+\left(A-A^{*}-A^{*}\ln\frac{A}{A^{*}}\right)\;+\left(I-I^{*}-I^{*}\ln\frac{I}{I^{*}}\right)+\left(H-H^{*}-H^{*}\ln\frac{H}{H^{*}}\right)+\left(R-R^{*}-R^{*}\ln\frac{R}{R^{*}}\right).$$

Differentiating ℒ with respect to time along the system trajectories:


$$\frac{dℒ}{dt}=\left(\frac{S-S^{*}}{S}\right)\dot{S}+\left(\frac{E-E^{*}}{E}\right)\dot{E}+\left(\frac{A-A^{*}}{A}\right)\dot{A}+\left(\frac{I-I^{*}}{I}\right)\dot{I}+\left(\frac{H-H^{*}}{H}\right)\dot{H}+\left(\frac{R-R^{*}}{R}\right)\dot{R}.$$

Substituting the expressions for $\dot{S},\dot{E},\dot{A},\dot{I},\dot{H},\dot{R}$ from the model (3.1):


$$\frac{dℒ}{dt}=\left(\frac{S-S^{*}}{S}\right)\left(\Lambda +\sigma R-\rho S(A+I)-\mu S\right)+\left(\frac{E-E^{*}}{E}\right)\left(\rho S(A+I)-(\beta_{1}+\beta_{2}+\mu )E\right)\;\;+\left(\frac{A-A^{*}}{A}\right)\left(\beta_{1}E-(\gamma_{1}+\gamma_{2}+\mu )A\right)+\left(\frac{I-I^{*}}{I}\right)\left(\beta_{2}E+\gamma_{2}A-(\alpha +\delta_{1}+\mu )I\right)\;\;+\left(\frac{H-H^{*}}{H}\right)\left(\alpha I-(\tau +\delta_{2}+\mu )H\right)+\left(\frac{R-R^{*}}{R}\right)\left(\gamma_{1}A+\tau H-(\sigma +\mu )R\right).$$

At the disease equilibrium N^*^, the following hold:


$$\left\{ \begin{array}{c} \Lambda +\sigma R^{*}=\rho S^{*}(A^{*}+I^{*})+\mu S^{*},\\ \rho S^{*}(A^{*}+I^{*})=(\beta_{1}+\beta_{2}+\mu )E^{*},\\ \beta_{1}E^{*}=(\gamma_{1}+\gamma_{2}+\mu )A^{*},\\ \beta_{2}E^{*}+\gamma_{2}A^{*}=(\alpha +\delta_{1}+\mu )I^{*},\\ \alpha I^{*}=(\tau +\delta_{2}+\mu )H^{*},\\ \gamma_{1}A^{*}+\tau H^{*}=(\sigma +\mu )R^{*}. \end{array}\right.$$

We can rewrite $\frac{dℒ}{dt}$ as:


$$\frac{dℒ}{dt}=\mu S^{*}\left(2-\frac{S}{S^{*}}-\frac{S^{*}}{S}\right)+\sigma R^{*}\left(1-\frac{S^{*}}{S}+\frac{R}{R^{*}}-\frac{R}{R^{*}}\frac{S^{*}}{S}\right)\;+\rho S^{*}(A^{*}+I^{*})\left[4-\frac{S^{*}}{S}-\frac{E}{E^{*}}\frac{S}{S^{*}}\frac{A+I}{A^{*}+I^{*}}-\frac{A^{*}}{A}\frac{E}{E^{*}}-\frac{I^{*}}{I}\left(\frac{E}{E^{*}}+\frac{A}{A^{*}}\right)\right]\;+\beta_{1}E^{*}\left(3-\frac{E^{*}}{E}-\frac{A}{A^{*}}\frac{E}{E^{*}}-\frac{A^{*}}{A}\right)\;+\beta_{2}E^{*}\left(3-\frac{E^{*}}{E}-\frac{I}{I^{*}}\frac{E}{E^{*}}-\frac{I^{*}}{I}\right)\;+\gamma_{2}A^{*}\left(3-\frac{A^{*}}{A}-\frac{I}{I^{*}}\frac{A}{A^{*}}-\frac{I^{*}}{I}\right)\;+\alpha I^{*}\left(3-\frac{I^{*}}{I}-\frac{H}{H^{*}}\frac{I}{I^{*}}-\frac{H^{*}}{H}\right)\;+\tau H^{*}\left(3-\frac{H^{*}}{H}-\frac{R}{R^{*}}\frac{H}{H^{*}}-\frac{R^{*}}{R}\right)\;+\gamma_{1}A^{*}\left(3-\frac{A^{*}}{A}-\frac{R}{R^{*}}\frac{A}{A^{*}}-\frac{R^{*}}{R}\right).$$

Now, using the inequality g(x)=1−x+lnx≤0 for all *x* > 0, with equality only at *x* = 1, we can rewrite each term. Therefore,


$$2-\frac{S}{S^{*}}-\frac{S^{*}}{S}=-\left[\left(\frac{S}{S^{*}}-1\right)+\left(\frac{S^{*}}{S}-1\right)\right]=-\left[g\left(\frac{S}{S^{*}}\right)+g\left(\frac{S^{*}}{S}\right)\right]\leq 0.$$

Similarly,


$$4-\frac{S^{*}}{S}-\frac{E}{E^{*}}\frac{S}{S^{*}}\frac{A+I}{A^{*}+I^{*}}-\frac{A^{*}}{A}\frac{E}{E^{*}}-\frac{I^{*}}{I}\left(\frac{E}{E^{*}}+\frac{A}{A^{*}}\right),$$

can be expressed as sums of g(x) functions, which are non-positive. All other terms follow the same pattern and are non-positive by the arithmetic mean-geometric mean inequality. Specifically, each term of the form:


$$n-\sum_{i=1}^{n}x_{i}\leq 0\;\text{when}\;\prod_{i=1}^{n}x_{i}=1,$$

which holds here by construction.

Thus, $\frac{dℒ}{dt}\leq 0$ for all (S,E,A,I,H,R)∈Ω, and $\frac{dℒ}{dt}=0$ if and only if


$$S=S^{*},\;E=E^{*},\;A=A^{*},\;I=I^{*},\;H=H^{*},\;R=R^{*}.$$

By LaSalle’s Invariance Principle, the endemic equilibrium N^*^ is globally asymptotically stable in Ω when ${ℛ}_{0}>1$. □

## 5 Forward bifurcation

This section provides a comprehensive forward bifurcation analysis of the given epidemiological model using center manifold theory [28]. We follow the approach outlined by Castillo-Chavez and Song [29]. It is noteworthy that the following study is analogous to that presented in [20].

Following Theorem 4.1 in [29], we analyze the bifurcation at ${ℛ}_{0}=1$ (where ${ℛ}_{0}$ is defined in (4.1)). Let the transmission rate from susceptible to exposed, denoted by ρ, be the bifurcation parameter with a critical value given by:


$$\rho^{*}=\frac{k_{1}k_{2}k_{3}}{S^{0}(\beta_{1}k_{3}+\beta_{2}k_{2}+\beta_{1}\gamma_{2})}.$$

The Jacobian $J({𝒟}_{0})$ at $\rho =\rho^{*}$ has a right eigenvector $𝐰=(w_{1},w_{2},w_{3},w_{4},w_{5},w_{6}{)}^{T}$ satisfying the condition $J({𝒟}_{0})𝐰=0$, where the system is derived from the Jacobian matrix evaluated at the disease-free equilibrium (DFE) ${𝒟}_{0}=(S_{0},0,0,0,0,0)$, with $S_{0}=\frac{\Lambda }{\mu }$. The right eigenvector 𝐰 corresponds to perturbations in the directions of the states S,E,A,I,H,R.

The Jacobian matrix $J({𝒟}_{0})$ for the given model is:


$$J({𝒟}_{0})=(\begin{array}{cccccc} -\mu & 0 & -\rho^{*}S_{0} & -\rho^{*}S_{0} & 0 & \sigma \\ 0 & -k_{1} & \rho^{*}S_{0} & \rho^{*}S_{0} & 0 & 0\\ 0 & \beta_{1} & -k_{2} & 0 & 0 & 0\\ 0 & \beta_{2} & \gamma_{2} & -k_{3} & 0 & 0\\ 0 & 0 & 0 & \alpha & -k_{4} & 0\\ 0 & 0 & \gamma_{1} & 0 & \tau & -(\sigma +\mu ) \end{array}),$$

where, $k_{1}=\beta_{1}+\beta_{2}+\mu ,\;k_{2}=\gamma_{1}+\gamma_{2}+\mu ,\;k_{3}=\alpha +\delta_{1}+\mu ,\;k_{4}=\tau +\delta_{2}+\mu$.

Multiplying $J({𝒟}_{0})$ by 𝐰 gives the system:


$$\left\{ \begin{array}{cc} -\mu w_{1}-\rho^{*}S_{0}w_{3}-\rho^{*}S_{0}w_{4}+\sigma w_{6}=0\; & \text{(}S\text{-equation)}\\ -k_{1}w_{2}+\rho^{*}S_{0}w_{3}+\rho^{*}S_{0}w_{4}=0\; & \text{(}E\text{-equation)}\\ \beta_{1}w_{2}-k_{2}w_{3}=0\; & \text{(}A\text{-equation)}\\ \beta_{2}w_{2}+\gamma_{2}w_{3}-k_{3}w_{4}=0\; & \text{(}I\text{-equation)}\\ \alpha w_{4}-k_{4}w_{5}=0\; & \text{(}H\text{-equation)}\\ \gamma_{1}w_{3}+\tau w_{5}-(\sigma +\mu )w_{6}=0\; & \text{(}R\text{-equation)} \end{array}\right.$$

At the disease-free equilibrium (DFE), all infected compartments (E,A,I,H) are zero. In the linearized analysis for eigenvectors, the terms $\sigma w_{6}$ is considered second-order effects (products of small perturbations) and are thus neglected. Consequently, the *S*-equation reduces to $-\mu w_{1}-\rho^{*}S_{0}(w_{3}+w_{4})=0$, while the other equations remain unchanged.

This yields the system:


$$\left\{ \begin{array}{c} -\mu w_{1}-\rho^{*}S_{0}(w_{3}+w_{4})=0,\\ \rho^{*}S_{0}(w_{3}+w_{4})-k_{1}w_{2}=0,\\ \beta_{1}w_{2}-k_{2}w_{3}=0,\\ \beta_{2}w_{2}+\gamma_{2}w_{3}-k_{3}w_{4}=0,\\ \alpha w_{4}-k_{4}w_{5}=0,\\ \gamma_{1}w_{3}+\tau w_{5}-(\sigma +\mu )w_{6}=0. \end{array}\right.$$

Solving sequentially, we have the following relationships: From the equation $\beta_{1}w_{2}\;-\;k_{2}w_{3}=0$, we obtain $w_{3}=\frac{\beta_{1}}{k_{2}}w_{2}$. Next, from $\beta_{2}w_{2}+\gamma_{2}w_{3}-k_{3}w_{4}=0$, we find $w_{4}=\frac{\beta_{2}k_{2}+\beta_{1}\gamma_{2}}{k_{2}k_{3}}w_{2}$. The third equation, $\rho^{*}S_{0}(w_{3}+w_{4})-k_{1}w_{2}=0$, is consistent by the definition of $\rho^{*}$. From the equation $-\mu w_{1}-\rho^{*}S_{0}(w_{3}+w_{4})=0$, we derive $w_{1}=-\frac{\rho^{*}S_{0}}{\mu }(w_{3}+w_{4})$. Furthermore, from $\alpha w_{4}-k_{4}w_{5}=0$, we obtain $w_{5}=\frac{\alpha }{k_{4}}w_{4}$. Finally, from the last equation, we have $w_{6}=\frac{\gamma_{1}w_{3}+\tau w_{5}}{\sigma +\mu }$.

We can set *w*_2_ = 1 for normalization, giving:


$$𝐰=(\begin{array}{c} -\frac{\rho^{*}S_{0}}{\mu }\left(\frac{\beta_{1}}{k_{2}}+\frac{\beta_{2}k_{2}+\beta_{1}\gamma_{2}}{k_{2}k_{3}}\right)\\ 1\\ \frac{\beta_{1}}{k_{2}}\\ \frac{\beta_{2}k_{2}+\beta_{1}\gamma_{2}}{k_{2}k_{3}}\\ \frac{\alpha (\beta_{2}k_{2}+\beta_{1}\gamma_{2})}{k_{2}k_{3}k_{4}}\\ \frac{\gamma_{1}\beta_{1}/k_{2}+\tau \alpha (\beta_{2}k_{2}+\beta_{1}\gamma_{2})/(k_{2}k_{3}k_{4})}{\sigma +\mu } \end{array}).$$

The left eigenvector $𝐯=(v_{1},v_{2},v_{3},v_{4},v_{5},v_{6})$ satisfies $𝐯J({𝒟}_{0})=0$:


$$\left\{ \begin{array}{c} -\mu v_{1}=0,\\ \rho^{*}S_{0}v_{2}-(\beta_{1}+\beta_{2}+\mu )v_{2}=0,\\ \rho^{*}S_{0}v_{2}+\beta_{1}v_{3}-(\gamma_{1}+\gamma_{2}+\mu )v_{3}+\gamma_{1}v_{6}=0,\\ \rho^{*}S_{0}v_{2}+\beta_{2}v_{3}+\gamma_{2}v_{4}-(\alpha +\delta_{1}+\mu )v_{4}+\alpha v_{5}=0,\\ \tau v_{6}-(\tau +\delta_{2}+\mu )v_{5}=0,\\ \sigma v_{1}+\sigma v_{6}-(\sigma +\mu )v_{6}=0. \end{array}\right.$$

Solving the equations sequentially, we find that $v_{1}=0$ from the first equation, while the second equation indicates that $v_{2}$ is free, so we set $v_{2}=1$. From the last equation, it follows that $v_{6}=0$, and the fifth equation gives us $v_{5}=0$. From the third equation, we express $v_{3}$ as $v_{3}=\frac{\rho^{*}S_{0}+\beta_{1}v_{3}-k_{2}v_{3}}{k_{2}}\Rightarrow v_{3}=\frac{\rho^{*}S_{0}}{k_{2}-\beta_{1}}$. Finally, from the fourth equation, we find $v_{4}=\frac{\rho^{*}S_{0}+\beta_{2}v_{3}}{k_{3}-\gamma_{2}}$. Thus,


$$𝐯=(\begin{array}{cccccc} 0, & 1, & \frac{\rho^{*}S_{0}}{k_{2}-\beta_{1}}, & \frac{\rho^{*}S_{0}+\beta_{2}\frac{\rho^{*}S_{0}}{k_{2}-\beta_{1}}}{k_{3}-\gamma_{2}}, & 0, & 0 \end{array}).$$

Following [29], we compute:


$$a=\sum_{k,i,j=1}^{6}v_{k}w_{i}w_{j}\frac{\partial^{2}f_{k}}{\partial x_{i}\partial x_{j}}({𝒟}_{0},\rho^{*}),\;b=\sum_{k,i=1}^{6}v_{k}w_{i}\frac{\partial^{2}f_{k}}{\partial x_{i}\partial \rho }({𝒟}_{0},\rho^{*}).$$

The non-zero second derivatives are:


$$\frac{\partial^{2}f_{1}}{\partial S\partial A}=\frac{\partial^{2}f_{1}}{\partial A\partial S}=-\rho^{*},\;\frac{\partial^{2}f_{1}}{\partial S\partial I}=\frac{\partial^{2}f_{1}}{\partial I\partial S}=-\rho^{*},$$

$$\frac{\partial^{2}f_{2}}{\partial S\partial A}=\frac{\partial^{2}f_{2}}{\partial A\partial S}=\rho^{*},\;\frac{\partial^{2}f_{2}}{\partial S\partial I}=\frac{\partial^{2}f_{2}}{\partial I\partial S}=\rho^{*}.$$

Thus, $a=2v_{2}(w_{1}w_{3}\rho^{*}+w_{1}w_{4}\rho^{*})-2v_{1}(w_{1}w_{3}\rho^{*}+w_{1}w_{4}\rho^{*})$. Since $v_{1}=0$ and $v_{2}=1$, we obtain


$$a=2\rho^{*}w_{1}(w_{3}+w_{4})<0\;(\text{since }w_{1}<0\text{ and }w_{3},w_{4}>0).$$

The non-zero derivatives are $\frac{\partial^{2}f_{2}}{\partial E\partial \rho }=S_{0}(w_{3}+w_{4})$. Thus, $b=v_{2}S_{0}(w_{3}+w_{4})>0$.

Since *a* < 0 and *b* > 0, the system exhibits a forward bifurcation at ${ℛ}_{0}=1$ [29]. This implies that for ${ℛ}_{0}<1$, only the disease-free equilibrium (DFE) exists and is stable; at ${ℛ}_{0}=1$, the DFE loses stability; and for ${ℛ}_{0}>1$, a stable endemic equilibrium emerges.

## 6 Parameter estimation, model fitting and sensitivity analysis

In this section, we examine the parameter estimation and conduct a sensitivity analysis.

### 6.1 Parameter estimation

Parameter estimation is carried out to calibrate the SEAIHR model (3.1) of Nipah virus transmission against available epidemiological data [17]. The unknown parameters are determined by minimizing the discripency between reported case counts and model predictions using a nonlinear least-squares approach [30–32]. The objective function is expressed as


$$J(\Theta )=\sum_{t=1}^{n}{\left(Y(t)-\hat{Y}(t;\Theta )\right)}^{2},$$

where Y(t) represents the observed data at time t, $\hat{Y}(t;\Theta )$ denotes the model output for the parameter set Θ, and n is the total number of observations. The optimal parameter vector is then obtained as


$$\Theta^{*}=\arg\min_{\Theta }J(\Theta ).$$

The estimated values are presented in Table 1, ensuring that the model dynamics are well calibrated with observed Nipah virus data.

### 6.2 Model fitting and residual analysis

Model parameters are estimated using least-squares fitting to Nipah virus data (2001-2025) [33]. Two fitting scenarios based on different values of the effective contact rate *ρ* are considered, with all other parameters fixed as in Table 1.

**Fig 4:**
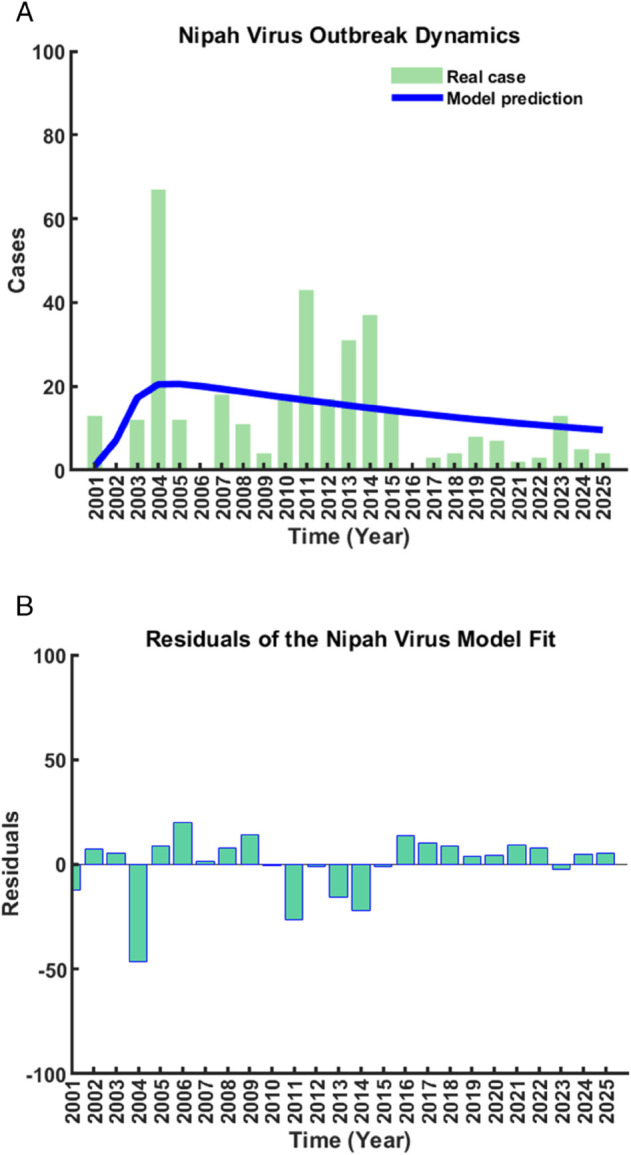
Model fitting and residual analysis for the Nipah virus outbreak with ρ=0.45 (a) Comparison between reported cases and model-predicted cases; (b) Corresponding residuals.

Using an effective contact rate of ρ=0.45, the model achieves strong agreement with the reported Nipah virus cases over the study period (Fig 4(a)). The fitted trajectory closely reproduces the observed outbreak progression, including the early growth phase, the mid-2011s peak, and the subsequent decline. A sudden surge observed around 2004 is partially underestimated, reflecting an abrupt outbreak event that is difficult to capture within a smooth modeling framework; however, other major peaks are well reproduced. Residuals are predominantly centered around zero, with most deviations confined within ±15 cases, indicating a well-balanced and unbiased fit (Fig 4(b)).

**Fig 5:**
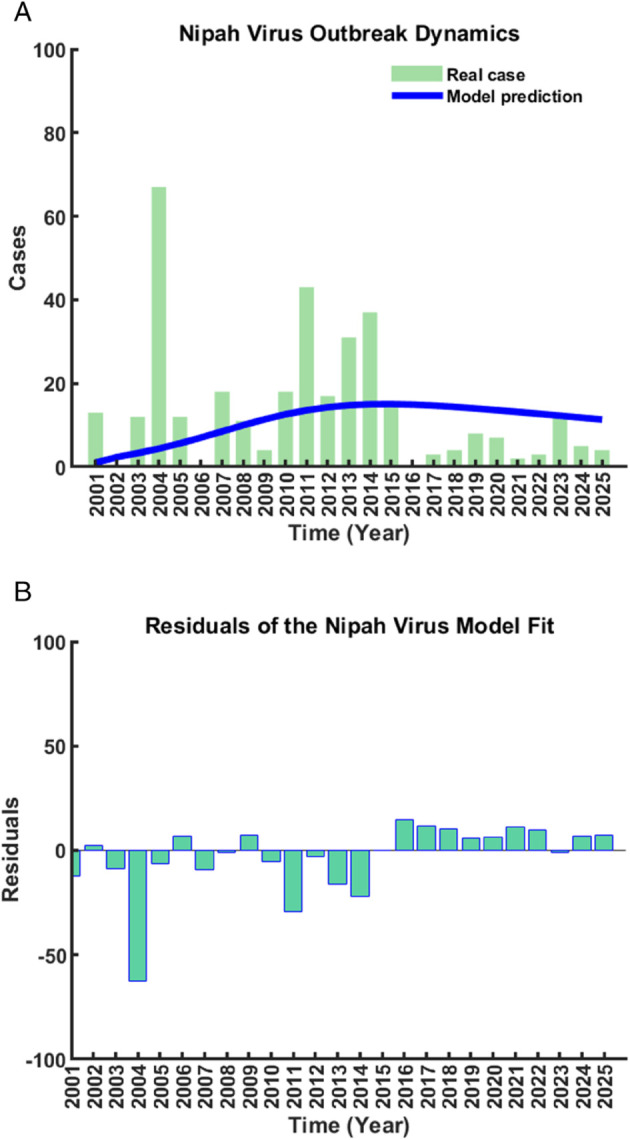
Model fitting and residual analysis for the Nipah virus outbreak with ρ=0.10 (a) Comparison between reported cases and model-predicted cases; (b) Corresponding residuals.

In contrast, adopting a lower effective contact rate (ρ=0.10) results in reduced agreement with the observed data (Fig 5(a)). While the overall outbreak trend is preserved, discrepancies increase during periods of elevated transmission, producing larger and more dispersed residuals, with several exceeding ±20 cases (Fig 5(b)). This comparison highlights the sensitivity of model performance to the effective contact rate.

### 6.3 Sensitivity analysis

Sensitivity analysis is essential for mathematical models as it identifies which parameters significantly influence outcomes, enhancing our understanding of model behavior. It assesses the robustness of the model by determining how small changes in parameters affect results, thereby guiding decision-making and policy formulation. Additionally, sensitivity analysis quantifies uncertainty in predictions and aids in model validation by comparing outputs with real-world data. Ultimately, it provides insights that can optimize parameter values, improving the model’s accuracy and applicability across various fields.

In this section, we conduct a sensitivity analysis of the model described by Eq (3.1).

The normalized sensitivity indices were calculated using the following formula

$$\Gamma_{p}^{{ℛ}_{0}}=\frac{\partial {ℛ}_{0}}{\partial p}\times \frac{p}{{ℛ}_{0}},$$ (3)

where ${ℛ}_{0}$ represents the basic reproduction number and *p* denotes a model parameter. Applying the approach given in Eq (3), we obtain the sensitivity indices presented in Table 2.

**Table 2:** Sensitivity indices for each parameter.

Parameter	Sensitivity Index $\Gamma_{p}^{{ℛ}_{0}}$
Λ	1.0000
*ρ*	1.0000
*μ*	–1.0175
$\beta_{1}$	0.1328
$\beta_{2}$	–0.1268
$\gamma_{1}$	–0.2815
$\gamma_{2}$	–0.1573
*α*	–0.3394
$\delta_{1}$	–0.2104
*σ*	0.0000
*τ*	0.0000
$\delta_{2}$	0.0000

**Fig 6:**
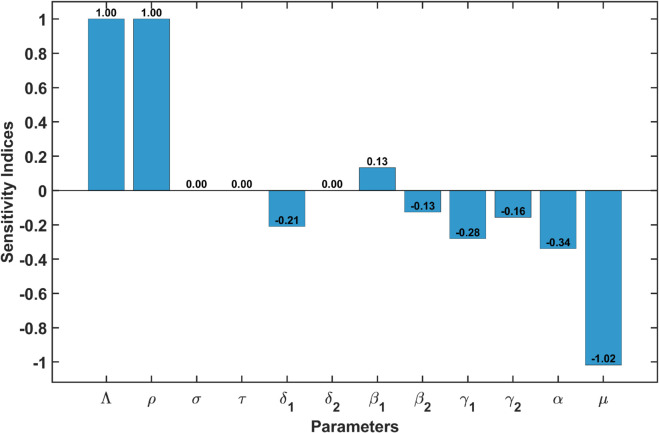
Bar chart displaying the sensitivity indices for various parameters.

The bar diagram in Fig 6 displays the outcomes of the sensitivity analysis performed on the model parameters.

The sensitivity indices provide several important insights regarding the influence of various parameters on the basic reproduction number ${ℛ}_{0}$:

1. **Dominant Parameters**: The parameters Λ (the recruitment rate) and ρ (the transmission rate) exhibit a sensitivity index of 1.0000. This indicates that a unit increase in either parameter will result in a proportional increase in ${ℛ}_{0}$. Consequently, these parameters are critical in determining the potential for disease transmission.
2. **Negative Impact of Mortality**: The sensitivity index for μ (the natural mortality rate) is –1.0175. This finding suggests that an increase in mortality has a considerable negative effect on ${ℛ}_{0}$, implying that higher mortality rates effectively diminish the transmission potential of the disease.
3. **Minor Contributions**: The parameters $\beta_{1}$ (the transmission rate for the first route of infection) and $\beta_{2}$ (the transmission rate for the second route of infection) have sensitivity indices of 0.1328 and –0.1268, respectively. Although these values indicate some degree of influence on ${ℛ}_{0}$, their impact is significantly less pronounced compared to Λ and ρ.
4. **Negative Indices**: Other parameters such as $\gamma_{1}$, $\gamma_{2}$, α, and $\delta_{1}$ also demonstrate negative sensitivity indices. This indicates that increases in these parameters will lead to a reduction in ${ℛ}_{0}$. Therefore, interventions that target these parameters could effectively lower disease transmission.
5. **Negligible Effects**: The parameters σ, τ, and $\delta_{2}$ exhibit sensitivity indices of 0.0000, signifying that variations in these parameters do not significantly affect ${ℛ}_{0}$.

## 7 Numerical simulation

Numerical simulations are conducted to investigate the transmission dynamics of Nipah virus under the proposed model (3.1), providing insights into epidemic progression and the potential effects of key transmission parameters.

**Fig 7:**
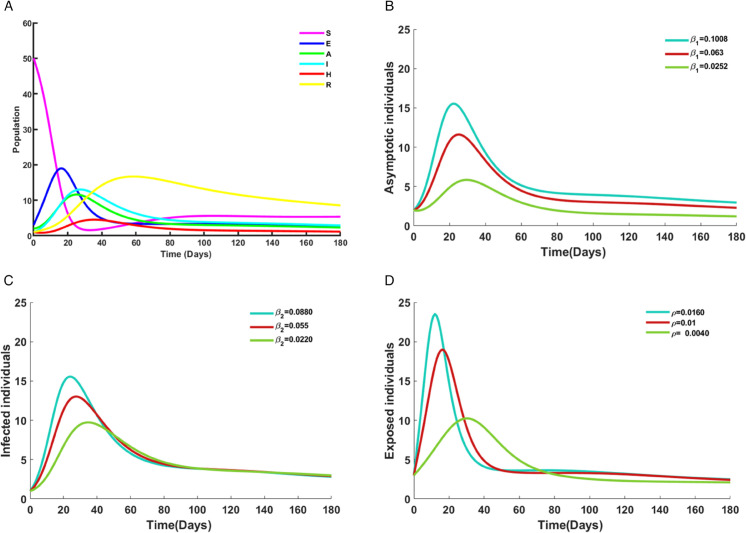
Solution trajectories of the model (1), (a) All compartments over time; (b) Impact of varying $\beta_{1}$ on Asymptotic individuals; (c) Impact of varying $\beta_{2}$ on Infected individuals; (d) Impact of varying *ρ* on Exposed individuals.

Fig 7(a) shows the time evolution of the Susceptible (*S*), Exposed (*E*), Asymptomatic (*A*), Infected (*I*), Hospitalized (*H*), and Recovered (*R*) populations over 180 days. The susceptible group declines rapidly from 50 to 3 within the first 30 days due to new exposures. The exposed class peaks at 19 on day 20 and declines to near zero by day 100. Asymptomatic infections rise to 11.5 by day 22 before steadily decreasing. Symptomatic infections peak at 14 around day 27 and then starts to decline. Hospitalizations reach 4.5 by day 34, while recoveries steadily accumulate, peaking near 18 by day 57 and stabilizing thereafter. These reflect the natural progression of Nipah virus infection, with a sharp depletion of susceptibles, delayed buildup of symptomatic and asymptomatic cases, and eventual recovery. These patterns emphasize the need for early interventions during the first 20-40 days, when transmission and hospitalizations are most intense.

Fig 7(b) depicts the effect of varying $\beta_{1}$ values (0.1008, 0.063, 0.0252) on the asymptomatic class. When $\beta_{1}=0.1008$, the asymptomatic population rises sharply to nearly 17 individuals by day 22, while for $\beta_{1}=0.063$ and $\beta_{1}=0.0252$, the peaks are lower at approximately 12 and 6, respectively. All curves eventually decline to a low steady state by day 140. The results indicate that higher transition into the asymptomatic class increases hidden transmission potential, emphasizing the challenge of controlling cases that often remain undetected.

Fig 7(c) illustrates the effect of different values of $\beta_{2}$(0.0880, 0.055, 0.0220) on the infected population. When $\beta_{2}=0.0880$, infections rise sharply to nearly 16 around day 21, whereas the peaks are lower at about 13 and 9 for $\beta_{2}=0.055$ and $\beta_{2}=0.0220$, respectively. Higher values of $\beta_{2}$ not only elevate the infection peak but also extend the infectious period, with cases remaining beyond day 100. This shows that more efficient progression from exposure to symptomatic infection amplifies epidemic severity, making rapid case detection and isolation crucial to reduce further spread.

Fig 7(d) presents the impact of varying *ρ* values (0.0160, 0.0100, 0.0040) on the exposed population. With ρ=0.0160, exposures peak near 23 individuals around day 17, whereas lower values of 0.0100 and 0.0040 result in peaks of about 18 and 10 respectively. Although the exposed population declines after day 80 in all scenarios, larger *ρ* values lead to a faster and more pronounced epidemic wave. This indicates that stronger transmission pressure from susceptibles to exposed individuals increases both the speed and scale of outbreaks, highlighting the need to curb contact and transmission early on.

Nipah virus dynamics are driven by transitions among Exposed, Asymptomatic, and Infected classes, where asymptomatic progression sustains hidden transmission, symptomatic progression increases disease burden, and higher exposure rates trigger faster and larger outbreaks. These findings highlight the critical importance of targeted public health interventions, including early detection of asymptomatic carriers, rapid isolation and treatment of symptomatic cases, and strategies that reduce contact opportunities to effectively limit Nipah virus transmission.

**Fig 8:**
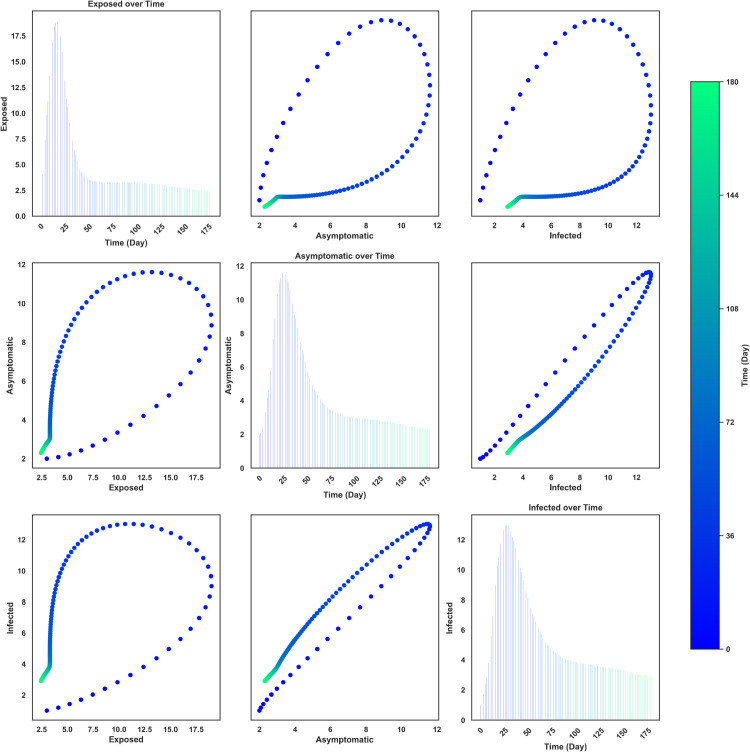
Pairwise relationships among Exposed, Asymptomatic, and Infected individuals over time. The color gradient represents the progression of Time (Day). Diagonal plots illustrate variable trends over time using bar plots, while off-diagonal plots depict bi-variate relationships colored by time.

Fig 8 presents the temporal and pairwise dynamics of Nipah virus transmission among Exposed (*E*), Asymptomatic (*A*), and Infected (*I*) individuals over 180 days. The diagonal bar plots show temporal trends, where the Exposed population initially rises rapidly, reaching a peak of approximately 19 individuals before steadily declining to near zero by day 180. Similarly, the Asymptomatic population peaks around 11.5, while the Infected class reaches approximately 14 individuals, both subsiding gradually after day 75 and 80 respectively. The epidemic trajectory is therefore characterized by a sharp early outbreak followed by stabilization at low levels.

The off-diagonal scatter plots reveal nonlinear bivariate relationships between the compartments. The *E*–*A* and *E*–*I* trajectories forms a distinctive loop, where the Exposed class peaks early at approximately 17 before beginning to decline, while asymptomatic (∼11.5) and infected (∼14) cases rise later due to the incubation period and then decrease. This sequential pattern underscores the role of exposure in driving delayed infection peaks and highlights the need for early interventions, such as rapid detection and isolation, to curb outbreak intensity.

The *A*–*I* relationship stands out as nearly linear and strongly positive, indicating that infections scale almost directly with the size of the asymptomatic class. Both compartments increase synchronously during the outbreak, peaking near 13 (on average) individuals, and decrease at similar rates afterward. This tight coupling suggests that the asymptomatic population plays a central role in fueling symptomatic infections, reflecting the epidemiological importance of silent transmission. Overall, the bivariate plots colored by time depict a wave-like outbreak, where exposure precedes and drives the rise of asymptomatic and symptomatic infections, followed by a synchronized decline across all compartments.

**Fig 9:**
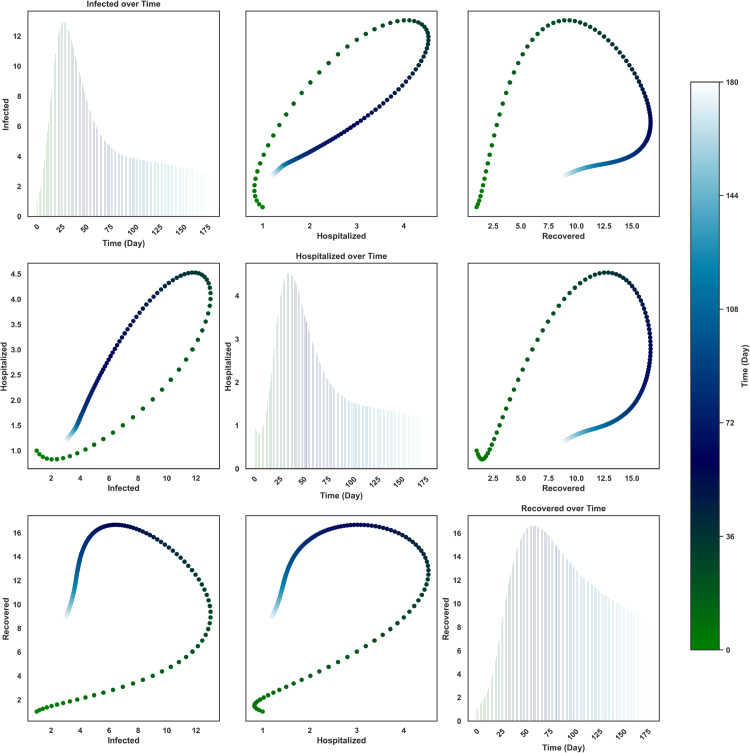
Pairwise relationships among Infected, Hospitalized, and Recovered individuals over time. The color gradient represents the progression of Time (Day). Diagonal plots illustrate variable trends over time using bar plots, while off-diagonal plots depict bi-variate relationships colored by time.

From Fig 9, the diagonal bar plots depict the time evolution of Infected (*I*), Hospitalized (*H*), and Recovered (*R*) individuals over a 180-day period. The Infected population peaks early at approximately 14, declining steadily afterward. The Hospitalized class rises more modestly, reaching a maximum of about 4.5, before gradually decreasing, in contrast, the Recovered population builds up more slowly but eventually reaches the highest value, peaking around 16, before stabilizing toward the end of the time horizon.

The off-diagonal scatter plots highlight pairwise relationships among the compartments, with points colored by time. The *I*–*H* trajectory forms a loop: infections increase rapidly to approximately 14, driving hospitalizations upward to 4.5, followed by a simultaneous decline. The *I*–*R* relationship shows a curved trajectory where recovered cases accumulate as infections increase, peaking at 16 once infections begin to decline. Similarly, the *H*–*R* relationship displays a nonlinear loop, where recovery continues to increase even as hospitalizations peak and subsequently decrease.

These dynamics indicate that hospitalizations and recoveries lag behind infections, consistent with the clinical course of Nipah virus, where symptomatic cases may progress to hospitalization before recovery. The trajectories suggest that the outbreak is driven by an early surge in infections, followed by delayed peaks in hospitalization and eventual accumulation of recoveries. The results emphasize the importance of rapid case management and timely hospital care to reduce the burden of infection and ensure higher recovery rates.

## 8 Optimal control model

We formulate an optimal control problem to minimize Nipah burden over a finite horizon [0, *T*]. The goal is to reduce infections, hospital occupancy, and deaths while accounting for implementation costs of interventions, by selecting measurable, bounded control functions $0\leq u_{i}(t)\leq 1.$ The resulting optimal policies balance early transmission suppression with case management, yielding time-varying strategies that lower peak prevalence and the final epidemic size. We introduce three time-dependent control variables into the system (3.1), namely

- *u*_1_(*t*): Effort to reduce contact (e.g., public health campaigns, movement restrictions). This directly reduces the force of infection.
- *u*_2_(*t*): Effort to isolate symptomatic infectious individuals (*I*). This moves them to the hospitalized class (*H*) faster, reducing their contact with the general population.
- *u*_3_(*t*): Effort to improve treatment for hospitalized individuals (*H*). This increases their recovery rate and reduces disease-induced mortality.

The controlled system of differential equations is given by

$$\left\{ \begin{array}{cc} & \dot{S}=\Lambda +\sigma R-\rho (1-u_{1}(t))S(A+I)-\mu S,\\ & \dot{E}=\rho (1-u_{1}(t))S(A+I)-(\beta_{1}+\beta_{2}+\mu )E,\\ & \dot{A}=\beta_{1}E-(\gamma_{1}+\gamma_{2}+\mu )A,\\ & \dot{I}=\beta_{2}E+\gamma_{2}A-(\alpha +\delta_{1}+\mu +u_{2}(t))I,\\ & \dot{H}=\alpha I+u_{2}(t)I-(\tau +\delta_{2}+\mu +u_{3}(t))H,\\ & \dot{R}=\gamma_{1}A+(\tau +u_{3}(t))H-(\sigma +\mu )R. \end{array}\right.$$ (4)

We define our objective functional to minimize the number of infectious individuals (both asymptomatic *A* and symptomatic *I*) and hospitalized individuals *H*, as well as the nonlinear cost of implementing the controls over a fixed time interval [0, *T*].


$$J(u_{1},u_{2},u_{3})=\int_{0}^{T}\left[w_{1}A(t)+w_{2}I(t)+w_{3}H(t)+\frac{1}{2}(c_{1}u_{1}^{2}(t)+c_{2}u_{2}^{2}(t)+c_{3}u_{3}^{2}(t))\right]dt.$$

Here, $w_{1},w_{2},w_{3}>0$ are weight constants that balance the importance of reducing the size of each infected compartment. Here, $c_{1},c_{2},c_{3}>0$ are weight constants related to the cost of implementing each control. The quadratic cost terms $\frac{1}{2}c_{i}u_{i}^{2}(t)$ are standard to model the notion that the cost of an intervention increases dramatically (e.g., due to resource limitations and efficiency losses) as the effort approaches its maximum.

Our goal is to find an optimal control triple ${𝐮}^{*}=(u_{1}^{*},u_{2}^{*},u_{3}^{*})$ such that


$$J({𝐮}^{*})=\min_{𝐮\in 𝒰}J(𝐮),$$

subject to the controlled system above, where the admissible control set 𝒰 is defined as


$$𝒰=\left\{(u_{1},u_{2},u_{3})|u_{i}(t)\text{ is Lebesgue measurable on }[0,T],\;0\leq u_{i}(t)\leq u_{i}^{\max}\leq 1,\;i=1,2,3\right\}.$$

The upper bounds $u_{i}^{\max}$ represent the maximum feasible implementation level of each control measure. This formulation allows for the analysis of the most effective and cost-efficient combination of non-pharmaceutical interventions and treatment improvements to manage a Nipah virus outbreak.

### 8.1 Existence of optimal control

The following Theorem 6 illustrates the existence of the optimal control model given in Eq (8.1). Note that the following proof is analogous to that in [[34], Theorem 6].

**Theorem 6.** *Consider the objective functional*


$$J(u_{1},u_{2},u_{3})=\int_{0}^{T}\left[w_{1}A(t)+w_{2}I(t)+w_{3}H(t)+\frac{1}{2}\left(c_{1}u_{1}^{2}(t)+c_{2}u_{2}^{2}(t)+c_{3}u_{3}^{2}(t)\right)\right]dt,$$

*subject to the controlled system given in Eq (8.1) with initial conditions*


$$S(0)=S_{0}\geq 0,\;E(0)=E_{0}\geq 0,\;A(0)=A_{0}\geq 0,\;I(0)=I_{0}\geq 0,\;H(0)=H_{0}\geq 0,\;R(0)=R_{0}\geq 0,$$

*and the admissible control set*


$$𝒰=\left\{(u_{1},u_{2},u_{3})|u_{i}(t)\in [0,u_{i}^{\max}],\;u_{i}^{\max}\leq 1,\;u_{i}\;is\;Lebesgue\;measurable\right\}.$$

*Then there exists an optimal control triple ${𝐮}^{*}=(u_{1}^{*},u_{2}^{*},u_{3}^{*})$ such that*


$$J({𝐮}^{*})=\min_{𝐮\in 𝒰}J(𝐮).$$

*Proof:* We verify the standard conditions for existence of optimal control following the approach in [35,36].

1. The control set 𝒰 is nonempty since constant functions *u*_*i*_(*t*) = 0 are Lebesgue measurable and satisfy $0\leq u_{i}(t)\leq u_{i}^{\max}$. The state system is Lipschitz continuous in the state variables and continuous in the controls, ensuring existence and uniqueness of solutions for any given control 𝐮∈𝒰.
2. The control set 𝒰 is convex. For any u,v∈𝒰 and ϕ∈[0,1], we have
  $$\phi u_{i}(t)+(1-\phi )v_{i}(t)\in [0,u_{i}^{\max}]\;\text{for all }t\in [0,T],$$
  and the convex combination remains Lebesgue measurable. The set is closed under uniform convergence since the limit of measurable functions with values in $\left[0,u_{i}^{\max}\right]$ is also measurable and takes values in the same interval.
3. Let $𝐱=(S,E,A,I,H,R{)}^{T}$. The state system can be written as
  $$\frac{d𝐱}{dt}=F(t,𝐱,𝐮),$$
  where *F* is continuous in all arguments. Since the total population *N* = *S* + *E* + *A* + *I* + *H* + *R* satisfies
  $$\frac{dN}{dt}=\Lambda -\mu N-\delta_{1}I-\delta_{2}H\leq \Lambda -\mu N,$$
  we have N(t)≤max{N(0),Λ/μ}, showing that state variables are bounded. The function *F* is Lipschitz continuous in **x** because all parameters are bounded and the nonlinear terms are polynomials.
4. The integrand $L(t,𝐱,𝐮)=w_{1}A+w_{2}I+w_{3}H+\frac{1}{2}\left(c_{1}u_{1}^{2}+c_{2}u_{2}^{2}+c_{3}u_{3}^{2}\right)$ is convex in **u**. The Hessian matrix with respect to **u** is
  $$\nabla_{𝐮}^{2}L=[\begin{array}{ccc} c_{1} & 0 & 0\\ 0 & c_{2} & 0\\ 0 & 0 & c_{3} \end{array}],$$
  which is positive definite since $c_{1},c_{2},c_{3}>0$. Therefore, *L* is strictly convex in **u**.
5. There exist constants *n*_1_ > 0, *n*_2_ > 0, and *n*_3_ > 1 such that
  $$L(t,𝐱,𝐮)\geq n_{1}|𝐮{|}^{n_{3}}-n_{2}.$$
  We have
  $$L\geq \frac{1}{2}\min\{c_{1},c_{2},c_{3}\}(u_{1}^{2}+u_{2}^{2}+u_{3}^{2})=n_{1}|𝐮{|}^{2},$$
  where $n_{1}=\frac{1}{2}\min\{c_{1},c_{2},c_{3}\}$ and *n*_3_ = 2. The term $w_{1}A+w_{2}I+w_{3}H$ is bounded below by 0 since A,I,H≥0.

Since all conditions are satisfied, by the Filippov-Cesari Theorem [35], there exists an optimal control triple ${𝐮}^{*}=(u_{1}^{*},u_{2}^{*},u_{3}^{*})$. □

### 8.2 Optimality conditions

The following Theorem 7 demonstrates the optimality conditions of the system described in Eq (8.1). Note that the proof is similar to that in [[34] Theorem 7, [37] Theorem 3, [38] Theorem 4].

**Theorem 7.** *Let ${𝐮}^{*}=(u_{1}^{*},u_{2}^{*},u_{3}^{*})$ be the optimal control and ${𝐱}^{*}=(S^{*},E^{*},A^{*},I^{*},H^{*},R^{*})$ be the corresponding state solution. Then there exist adjoint variables $\lambda_{S},\lambda_{E},\lambda_{A},\lambda_{I},\lambda_{H},\lambda_{R}$ satisfying the following system*


$$\frac{d\lambda_{S}}{dt}=-\frac{\partial ℋ}{\partial S}=\lambda_{S}\left[\rho (1-u_{1})(A+I)+\mu \right]-\lambda_{E}\rho (1-u_{1})(A+I),\;\frac{d\lambda_{E}}{dt}=-\frac{\partial ℋ}{\partial E}=\lambda_{E}(\beta_{1}+\beta_{2}+\mu )-\lambda_{A}\beta_{1}-\lambda_{I}\beta_{2},\;\frac{d\lambda_{A}}{dt}=-\frac{\partial ℋ}{\partial A}=-w_{1}+\lambda_{S}\rho (1-u_{1})S-\lambda_{E}\rho (1-u_{1})S+\lambda_{A}(\gamma_{1}+\gamma_{2}+\mu )-\lambda_{I}\gamma_{2}-\lambda_{R}\gamma_{1},\;\frac{d\lambda_{I}}{dt}=-\frac{\partial ℋ}{\partial I}=-w_{2}+\lambda_{S}\rho (1-u_{1})S-\lambda_{E}\rho (1-u_{1})S+\lambda_{I}(\alpha +\delta_{1}+\mu +u_{2})-\lambda_{H}(\alpha +u_{2}),\;\frac{d\lambda_{H}}{dt}=-\frac{\partial ℋ}{\partial H}=-w_{3}+\lambda_{H}(\tau +\delta_{2}+\mu +u_{3})-\lambda_{R}(\tau +u_{3}),\;\frac{d\lambda_{R}}{dt}=-\frac{\partial ℋ}{\partial R}=-\lambda_{S}\sigma +\lambda_{R}(\sigma +\mu ),$$

*with transversality conditions*


$$\lambda_{S}(T)=0,\;\lambda_{E}(T)=0,\;\lambda_{A}(T)=0,\;\lambda_{I}(T)=0,\;\lambda_{H}(T)=0,\;\lambda_{R}(T)=0.$$

*The optimal controls are characterized by*


$$u_{1}^{*}=\min\left\{u_{1}^{\max},\;\max\left\{0,\;\frac{\rho S(A+I)(\lambda_{E}-\lambda_{S})}{c_{1}}\right\}\right\},\;u_{2}^{*}=\min\left\{u_{2}^{\max},\;\max\left\{0,\;\frac{I(\lambda_{I}-\lambda_{H})}{c_{2}}\right\}\right\},\;u_{3}^{*}=\min\left\{u_{3}^{\max},\;\max\left\{0,\;\frac{H(\lambda_{H}-\lambda_{R})}{c_{3}}\right\}\right\}.$$

*Proof:* We apply Pontryagin’s Maximum Principle [39].

1. The Hamiltonian function is defined as
  $$ℋ=w_{1}A+w_{2}I+w_{3}H+\frac{1}{2}\left(c_{1}u_{1}^{2}+c_{2}u_{2}^{2}+c_{3}u_{3}^{2}\right)+\sum \lambda_{i}\frac{dx_{i}}{dt},$$
  where the sum is over all state variables. Explicitly
  $$ℋ=w_{1}A+w_{2}I+w_{3}H+\frac{1}{2}\left(c_{1}u_{1}^{2}+c_{2}u_{2}^{2}+c_{3}u_{3}^{2}\right)\;+\lambda_{S}\left[\Lambda +\sigma R-\rho (1-u_{1})S(A+I)-\mu S\right]+\lambda_{E}\left[\rho (1-u_{1})S(A+I)-(\beta_{1}+\beta_{2}+\mu )E\right]\;+\lambda_{A}\left[\beta_{1}E-(\gamma_{1}+\gamma_{2}+\mu )A\right]+\lambda_{I}\left[\beta_{2}E+\gamma_{2}A-(\alpha +\delta_{1}+\mu +u_{2})I\right]\;+\lambda_{H}\left[\alpha I+u_{2}I-(\tau +\delta_{2}+\mu +u_{3})H\right]+\lambda_{R}\left[\gamma_{1}A+(\tau +u_{3})H-(\sigma +\mu )R\right].$$
2. The adjoint equations are derived from
  $$\frac{d\lambda_{i}}{dt}=-\frac{\partial ℋ}{\partial x_{i}},\;\text{for }i\in \{S,E,A,I,H,R\}.$$
  For $\lambda_{S}$,
  $$\frac{d\lambda_{S}}{dt}=-\frac{\partial ℋ}{\partial S}\;=-\left[-\lambda_{S}\rho (1-u_{1})(A+I)-\lambda_{S}\mu +\lambda_{E}\rho (1-u_{1})(A+I)\right]\;=\lambda_{S}\left[\rho (1-u_{1})(A+I)+\mu \right]-\lambda_{E}\rho (1-u_{1})(A+I).$$
  The other adjoint equations are derived similarly by taking partial derivatives with respect to each state variable.
3. Since the objective functional does not have a terminal cost, the transversality conditions are $\lambda_{i}(T)=0\;\text{for all state variables}$.
4. The optimal controls are found by solving
  $$\frac{\partial ℋ}{\partial u_{j}}=0\;\text{for }j=1,2,3.$$
  For *u*_1_:
  $$\frac{\partial ℋ}{\partial u_{1}}=c_{1}u_{1}+\lambda_{S}\rho S(A+I)-\lambda_{E}\rho S(A+I)\;=c_{1}u_{1}+\rho S(A+I)(\lambda_{S}-\lambda_{E})=0,\;\Rightarrow u_{1}=\frac{\rho S(A+I)(\lambda_{E}-\lambda_{S})}{c_{1}}.$$
  Considering the bounds $0\leq u_{1}\leq u_{1}^{\max}$ gives the final characterization.
  Similarly, for *u*_2_ and *u*_3_, we obtain, $u_{2}=\frac{I(\lambda_{I}-\lambda_{H})}{c_{2}},\;\text{and}\;\;u_{3}=\frac{H(\lambda_{H}-\lambda_{R})}{c_{3}}.$

The bounded controls are obtained by projecting onto $\left[0,u_{i}^{\max}\right]$. □

### 8.3 Optimal control strategy

Optimal control plays a crucial role in formulating effective strategies for managing infectious diseases, developing treatment protocols, and optimizing resource allocation to mitigate both the spread and impact of epidemics [40–42]. In this section, we introduce the concepts of Fraction of Immunized Agents (FIA) and Final Epidemic Size (FES). The underlying ideas are adapted from [43].

#### 8.3.1 Fraction of Immunized Agents (FIA) and Final Epidemic Size (FES).

The impact of control measures on epidemic outcomes can be quantitatively assessed using two fundamental metrics: the Fraction of Immunized Agents (FIA) and the Final Epidemic Size (FES). These metrics provide a framework for evaluating the long-term effectiveness of the control strategies *u*_1_(*t*), *u*_2_(*t*), and *u*_3_(*t*).

**Definition 1.** *FIA refers to the proportion of the population that has acquired immunity to the disease by the end of the epidemic. This immunity is achieved through either recovery from infection. In the context of this model, it is computed at the final time,* *t* = *T* *as:*


$$\text{FIA}=\frac{R(T)+H(T)}{N},$$

*where,*

- *R(T) is the number of recovered individuals at time T,*
- *H(T) is the number of hospitalized individuals at time T (who are assumed to eventually recover and gain immunity), and*
- *N is the total constant population.*

The FIA metric is dynamically influenced by the waning-immunity rate *σ*, which governs the transition of recovered individuals back to the susceptible compartment. In the model (3.1), this is captured by the term σR in the susceptible equation:


$$\dot{S}=\Lambda +\sigma R-\rho S(A+I)-\mu S.$$

A higher *σ* accelerates the loss of immunity, thereby reducing the effective immune fraction over time and increasing the risk of secondary outbreaks. Consequently, control strategies that maximize FIA must not only promote recovery but also account for the durability of immunity. In practice, sustaining a high FIA may require complementary interventions such as repeat vaccination or immunity-boosting measures to offset the effects of waning immunity and maintain long-term outbreak control.

**Fig 10:**
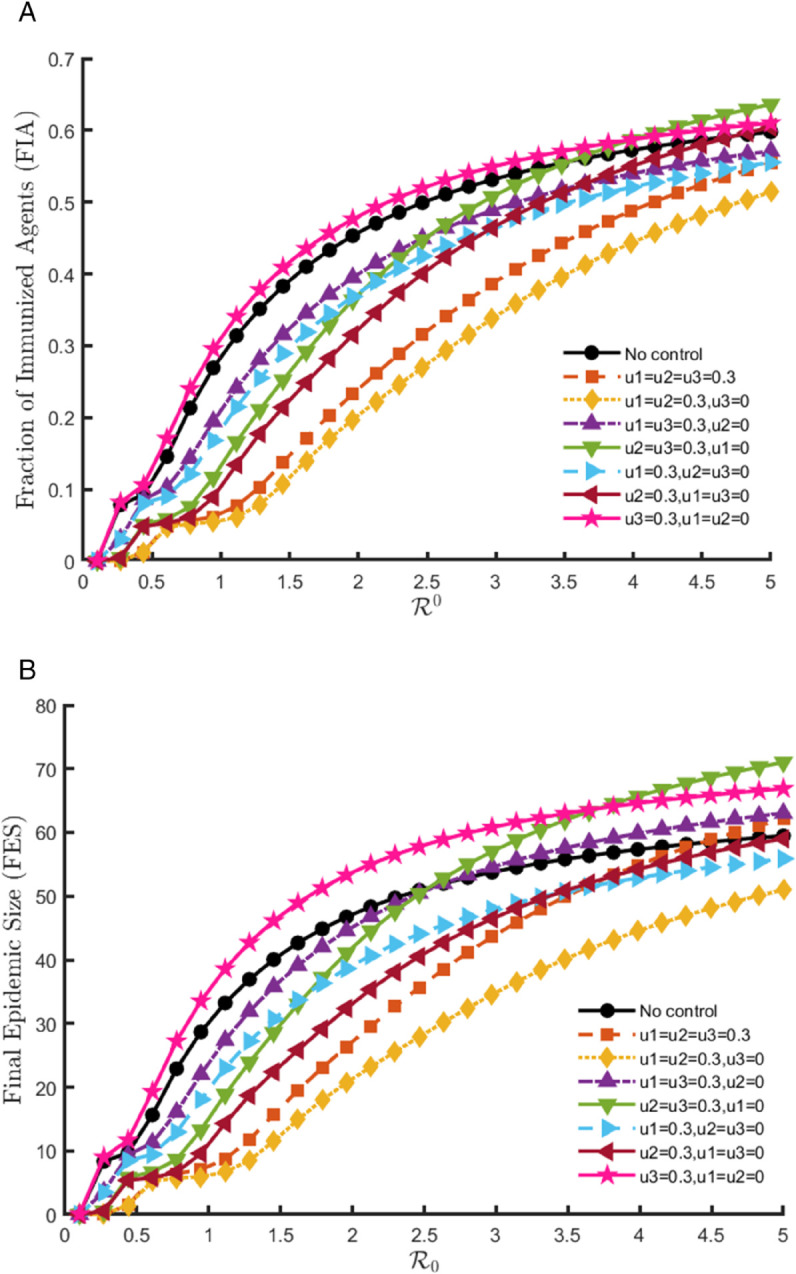
FIA and FES outcomes across varying basic reproduction numbers for the model (8.1), under moderate-intensity control ($u_{i}$ = 0.3).

The Fig 10 illustrates that at moderate control intensity (*u*_*i*_ = 0.3), the overall impact of interventions is limited, but the comparative patterns among different strategies remain informative. Among the individual interventions, isolation (*u*_2_ = 0.3) achieves modest reductions in both FIA (Fig 10(a)) and FES (Fig 10(b)), indicating some control of epidemic size while maintaining limited immunity buildup. Contact reduction alone (*u*_1_ = 0.3) produces minimal deviation from the no-control baseline, highlighting its weak effect at low intensity. Treatment alone (*u*_3_ = 0.3) instead increases both FIA and FES, since faster recovery without significant transmission reduction allows more infections to accumulate before epidemic decline, making it counterproductive as a single measure.

Pairwise strategies provide stronger effects than single interventions. The combination of contact reduction and treatment $\left(u_{1}=u_{3}=0.3,u_{2}=0\right)$ lowers FES relative to the no-control case by reducing exposure and hastening recovery, though its influence remains weaker than combinations involving isolation. Strategies such as $u_{1}=u_{2}=0.3,u_{3}=0$ or $u_{2}=u_{3}=0.3,u_{1}=0$ show clearer improvements, producing lower epidemic size while maintaining FIA at levels comparable to or below baseline. The integrated strategy where all three controls are applied simultaneously $\left(u_{1}=u_{2}=u_{3}=0.3\right)$ delivers the most consistent benefits under moderate intensity, pushing FES further down than any single or pairwise measure, while preserving a balanced immunity profile. Taken together, these results show that at low intensity, interventions are not sufficient to sharply alter epidemic dynamics, yet integrated approaches still outperform isolated measures and lay the groundwork for stronger suppression when scaled up.

**Remark 2.** *The FIA metric effectively captures the success of control strategies in enhancing population immunity, which is critical for long-term disease management.*

**Definition 2.** *FES indicates the total number of individuals who have been infected during the entire course of the epidemic. This includes those who are still infected, those who have recovered, and those who are hospitalized. It is calculated at the final time,* *t* = *T* *as:*


FES=I(T)+R(T)+H(T),

*where,*

- *I(T) denotes the number of infected individuals remaining at the conclusion of the simulation,*
- *R(T) signifies the total number of individuals who have successfully recovered, and*
- *H(T) indicates the total number of individuals who are hospitalized.*

**Fig 11:**
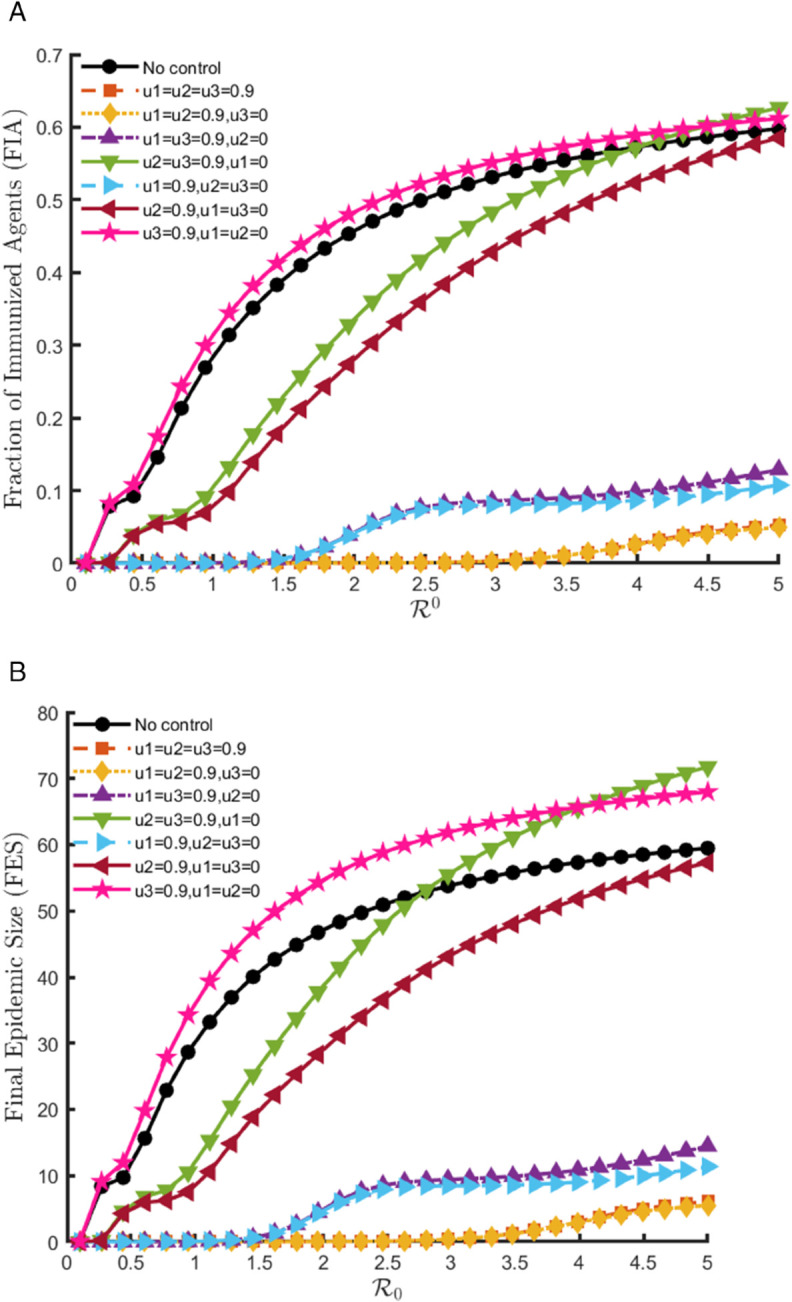
FIA and FES outcomes across varying basic reproduction numbers for the model (8.1), under high-intensity control ($u_{i}$ = 0.9).

At high control intensity (*u*_*i*_ = 0.9), the contrast between intervention types is more pronounced. Among the individual interventions, isolation (*u*_2_ = 0.9) provides robust epidemic suppression, substantially reducing FES while maintaining FIA at moderate levels, reflecting fewer infections but still some immunity gained through controlled recovery. Contact reduction (*u*_1_ = 0.9) also drives both FIA (Fig 11(a)) and FES (Fig 11(b)) to very low levels, indicating strong epidemic suppression, but as almost no immunity is generated, its benefit is fragile and heavily dependent on sustained restrictions. In contrast, treatment alone (*u*_3_ = 0.9) increases both FIA and FES above baseline, since immunity gains arise primarily from larger outbreaks, making it counterproductive as a standalone measure.

When interventions are combined, more nuanced patterns emerge. The pairwise strategy of contact reduction with treatment ($u_{1}=u_{3}=0.9,u_{2}=0$) lowers FES relative to baseline through reduced exposure and faster recovery, but without isolation its impact remains weaker than other combinations. By contrast, strategies involving isolation-such as $u_{1}=u_{2}=0.9,u_{3}=0$ or $u_{2}=u_{3}=0.9,u_{1}=0$ achieve sharper reductions in epidemic size while maintaining more stable FIA levels. The integrated scenario ($u_{1}=u_{2}=u_{3}=0.9$) produces the most favorable results overall, combining strong epidemic suppression with balanced immunity outcomes. These findings emphasize that while both contact reduction and treatment provide benefits, isolation remains central to effective epidemic control, and the most reliable outcomes arise from integrated interventions where all measures reinforce one another.

This analysis highlights how different control strategies influence the total number of cases over time. Each colored line corresponds to a specific combination of control parameters, demonstrating that higher intervention levels lead to a reduced final epidemic size.

**Remark 3.** *The FES metric provides critical insight into the overall burden of the epidemic, highlighting the effectiveness of control strategies in reducing the total number of cases.*

**Remark 4.** *The relationship between the basic reproduction number ${ℛ}_{0}$ and these two metrics is central to evaluating control efficacy. An effective control strategy will demonstrate a reduced FES across a range of ${ℛ}_{0}$ values, indicating its robustness in mitigating outbreaks of varying inherent transmissibility. Simultaneously, the same strategy will yield a higher FIA for a given ${ℛ}_{0}$, signifying its success in building population immunity through recovery, which is crucial for preventing future resurgence of the disease.*

## 9 Summary and discussion

This work presents a mathematical modeling framework to examine the transmission dynamics of Nipah virus (NiV) and evaluate potential intervention strategies. The proposed SEAIHR compartmental structure incorporates key epidemiological characteristics including asymptomatic transmission, hospitalization, and waning immunity. Analytical investigation established the fundamental epidemic threshold ${ℛ}_{0}$ and demonstrated global stability of the disease-free equilibrium when ${ℛ}_{0}\leq 1$, with a disease equilibrium becoming stable when ${ℛ}_{0}>1$. The identified forward bifurcation at ${ℛ}_{0}=1$ provides important insight into epidemic behavior, indicating that reducing the reproduction number below unity is essential for disease containment. Analysis of the forward bifurcation phenomenon revealed predictable transition behavior near the critical threshold ${ℛ}_{0}=1$, which carries significant implications for intervention design. Sensitivity analysis identified the recruitment rate and transmission rate as exerting the strongest influence on disease spread, emphasizing the importance of managing transmission dynamics. The optimal control component demonstrates how time-dependent interventions including contact reduction, case isolation, and enhanced treatment can be strategically implemented to minimize infection burden. Numerical simulations showed these optimized strategies substantially reduce the Final Epidemic Size (FES) while increasing the Fraction of Immunized Agents (FIA), with the FIA metric illustrating how control measures contribute to population-level immunity through recovery processes.

The findings provide valuable insights for disease management and intervention strategy development. The quantitative framework enables efficient resource allocation and strategic planning for outbreak response, while the model’s capacity to simulate various intervention scenarios through FES and FIA outcomes offers practical utility for regions where Nipah virus remains a persistent concern. The bifurcation analysis further enhances theoretical understanding of epidemic transition behavior, contributing to ongoing efforts to mitigate the impact of this serious pathogen.

## Supporting information

S1 File Supplementary file. (PDF)
