# Supplementary material for: Bifurcations and optimal control in Nipah virus epidemiology
Source: PLoS One. 2026 Mar 11;21(3):e0342764. doi: 10.1371/journal.pone.0342764 (PMC12978506; doi:10.1371/journal.pone.0342764)
Supplement: S1 File — (PDF) [file pone.0342764.s001.pdf]

Bifurcations and Optimal Control in Nipah Virus Epidemiology

Zasmin Haque [*1,4], Md. Mashih Ibn Yasin Adan[†2], Md. Sabab Zulfiker [‡3], Faizunnesa Khondaker[§1,5], and Md. Kamrujjaman [¶1]

[1]Department of Mathematics, University of Dhaka, Dhaka 1000, Bangladesh
[2]Department of Mathematics, Kishoreganj University, Kishoreganj, Bangladesh
[3]Department of Computer Science and Engineering, Kishoreganj University, Kishoreganj, Bangladesh
[4]Department of Mathematics, American International University-Bangladesh, Dhaka, Bangladesh
[5]Department of Mathematics, Jagannath University, Dhaka 1100, Bangladesh

Supplementary Results

This supplementary document contains a comprehensive analysis of the model solution for the mathematical framework outlined in the main manuscript.

A Mathematical Model

The model equations are given below

$$\begin{cases} \dot{S} = \Lambda + \sigma R - \rho S(A+I) - \mu S, \\ \dot{E} = \rho S(A+I) - (\beta_1 + \beta_2 + \mu)E, \\ \dot{A} = \beta_1 E - (\gamma_1 + \gamma_2 + \mu)A, \\ \dot{I} = \beta_2 E + \gamma_2 A - (\alpha + \delta_1 + \mu)I, \\ \dot{H} = \alpha I - (\tau + \delta_2 + \mu)H, \\ \dot{R} = \gamma_1 A + \tau H - (\sigma + \mu)R. \end{cases} \tag{A.1}$$

where all parameters are defined in Table A.1.

[*]Email: zasmin@aiub.edu
[†]Email: mdadan1081@gmail.com
[‡]Email: sabab.rumc@gmail.com
[§]Email: kh.faizunnesa@gmail.com
[¶]Corresponding author email: kamrujjaman@du.ac.bd

Table A.1: The biological implications of the parameters within the mathematical system (A.1) are of considerable significance.

**Notations**	**Overview**
Λ	Recruitment rate
ρ	Rate of transmission from susceptible to exposed
μ	Natural death rate
β_1	Transmission rate from exposed to asymptomatic individuals
β_2	Transmission rate from exposed to symptomatic individuals
γ_1	Proportion of recovery in asymptomatic population
γ_2	Proportion of recovery in symptomatic population
σ	Immunity loss rate among recovered individuals
α	Transition rate of symptomatic individuals to hospitalization
δ_1	Disease-related mortality rate among infected population
τ	Rate of recovery among hospitalized patients
δ_2	Disease-related mortality rate from the hospitalized class

B Analysis of Model Solution

The solutions of the dynamic system must be positive and bounded for all time values to ensure the biological validity of the mathematical model. The following lemmas provide proof of the positivity and boundedness of these solutions.

B.1 Positivity

In this section, we demonstrate the positivity of the model solution. It is important to note that the proof closely resembles that in [[1], Lemma 1].

Lemma 1. *Given the initial conditions $S(0) > 0$, $E(0) > 0$, $A(0) > 0$, $I(0) > 0$, $H(0) > 0$, $R(0) > 0$, and $t_0 > 0$, it follows that $\forall\ t \in [0, t_0]$, the functions $S(t)$, $E(t)$, $A(t)$, $I(t)$, $H(t)$, and $R(t)$ remain positive in $\mathbb{R}^6_+$.*

Proof. We assume that all parameters within the model are positive, which enables us to define a lower bound for each equation in (A.1) as follows:

$$\dot{S} > -(\rho(A+I)+\mu)S, \quad \dot{E} > -(\beta_1+\beta_2+\mu)E, \quad \dot{A} > -(\gamma_1+\gamma_2+\mu)A,$$
$$\dot{I} > -(\alpha+\delta_1+\mu)I, \quad \dot{H} > -(\tau+\delta_2+\mu)H, \quad \dot{R} > -(\sigma+\mu)R.$$

Solving each of the above inequalities separately gives:

$$S(t) > S(0)\exp(-\mu t - \rho\int(A+I)tdt) > 0, \quad E(t) > E(0)\exp(-(\beta_1+\beta_2+\mu)t) > 0,$$
$$A(t) > A(0)\exp(-(\gamma_1+\gamma_2+\mu)t) > 0, \quad I(t) > I(0)\exp(-(\alpha+\delta_1+\mu)t) > 0,$$
$$H(t) \geq H(0)\exp(-(\tau+\delta_2+\mu)t) > 0, \quad R(t) \geq R(0)\exp(-(\sigma+\mu)t) > 0.$$

As a result, for every t in the interval $[0, t_0]$, the functions $S(t)$, $E(t)$, $I(t)$, $A(t)$, $H(t)$, and $R(t)$ remain positive in $\mathbb{R}^6_+$. □

B.2 Boundedness

In this section, we establish the boundedness of the model solution. It is noteworthy that the proof is similar to that presented in [[2] Theorem 2, [1] Lemma 2].

Lemma 2. *For the functions $S(t)$, $E(t)$, $A(t)$, $I(t)$, $H(t)$, and $R(t)$ defined in (A.1), there are positive constants S_m, E_m, A_m, I_m, H_m, and R_m. These constants satisfy the following conditions:*

$$\lim_{t\to\infty} \sup S(t) \leq S_m, \quad \lim_{t\to\infty} \sup E(t) \leq E_m, \quad \lim_{t\to\infty} \sup A(t) \leq A_m,$$

$$\lim_{t\to\infty} \sup I(t) \leq I_m, \quad \lim_{t\to\infty} \sup H(t) \leq H_m, \quad \lim_{t\to\infty} \sup R(t) \leq R_m.$$

This is true for all t in the interval $[0, t_0]$, where $t_0 > 0$.

Proof. To establish boundedness, we begin by summing all the equations in the model (A.1), leading to the expression

$$\frac{dN}{dt} = \Lambda - \mu N - (\delta_1 I + \delta_2 H) \leq \Lambda - \mu N.$$

From this, it follows that

$$\frac{dN}{dt} \leq 0 \quad \text{if } N \geq \frac{\Lambda}{\mu}.$$

By addressing the inequality $\frac{dN}{dt} \leq \Lambda - \mu N$ and applying Gronwall's inequality, we can derive

$$N(t) \leq \frac{\Lambda}{\mu} + \left(N(0) - \frac{\Lambda}{\mu}\right) e^{-\mu t}.$$

As t approaches infinity, we conclude that

$$\lim_{t\to\infty} \sup N(t) \leq \frac{\Lambda}{\mu}.$$

By selecting $S_m = E_m = A_m = I_m = H_m = R_m = \frac{\Lambda}{\mu}$ with $t_0 > 0$, we can assert that $S(t)$, $E(t)$, $I(t)$, $A(t)$, $H(t)$, and $R(t)$ remain bounded, since

$$S(t), E(t), A(t), I(t), H(t), R(t) \leq N(t) \leq \frac{\Lambda}{\mu}.$$

Consequently, the region

$$\Omega = \left\{(S, E, A, I, H, R) \in \mathbb{R}^6_+ : S(t) + E(t) + A(t) + I(t) + H(t) + R(t) \leq \frac{\Lambda}{\mu}\right\}, \tag{B.1}$$

represents a positively invariant region for model (A.1). Moreover, if $N(0) > \frac{\Lambda}{\mu}$, then either the solution of (A.1) enters Ω within a finite time frame, or $N(t)$ approaches $\frac{\Lambda}{\mu}$ asymptotically. Therefore, the region Ω attracts all solutions of (A.1) within $\mathbb{R}^6_+$. □

B.3 Existence and Uniqueness

In this section, we establish the existence and uniqueness of the solution for the model. It is worth mentioning that the proof is similar to that presented in [[2] Theorem 1, [1] Lemma 3].

Lemma 3. *In the context of model (A.1), when the initial conditions satisfy* $S(0) > 0$, $E(0) > 0$, $I(0) > 0$, $A(0) > 0$, $H(0) > 0$, *and* $R(0) > 0$, *along with* $t_0 > 0$, *it follows that the solutions* $S(t)$, $E(t)$, $I(t)$, $A(t)$, $H(t)$, *and* $R(t)$ *will be present in* $\mathbb{R}^6_+$ *for all* $t \in \mathbb{R}$.

Proof. The model (A.1) can be represented as

$$\dot{y} = g(y),$$

where, y comprises the state variables:

$$\dot{y} = \begin{pmatrix} S(t) \\ E(t) \\ A(t) \\ I(t) \\ H(t) \\ R(t) \end{pmatrix}; \quad g(y) = \begin{pmatrix} \Lambda + \sigma R - \rho S(A+I) - \mu S \\ \rho S(A+I) - (\beta_1 + \beta_2 + \mu)E \\ \beta_1 E - (\gamma_1 + \gamma_2 + \mu)A \\ \beta_2 E + \gamma_2 A - (\alpha + \delta_1 + \mu)I \\ \alpha I - (\tau + \delta_2 + \mu)H \\ \gamma_1 A + \tau H - (\sigma + \mu)R \end{pmatrix}.$$

Given that g possesses a continuous first derivative in $\mathbb{R}^6_+$, it is classified as locally Lipschitz. Consequently, according to the well-known fundamental existence and uniqueness theorem [3] and the previously established Lemmas 1 and 2, there exists a unique, positive, and bounded solution for the system of differential equations (A.1) within $\mathbb{R}^6_+$. □

References

[1] Deressa CT, Duressa GF. Modeling and optimal control analysis of transmission dynamics of COVID-19: The case of Ethiopia. Alexandria Engineering Journal. 2021 Feb 1;60(1):719-32.

[2] Kifle ZS, Obsu LL. Mathematical modeling for COVID-19 transmission dynamics: A case study in Ethiopia. Results in Physics. 2022 Mar 1;34:105191.

[3] Perko L. Differential equations and dynamical systems. Springer Science & Business Media; 2013 Nov 21.
